# High‐Energy‐Density Redox Flow Batteries: Mechanisms, Design Strategies, and Recent Progress

**DOI:** 10.1002/chem.202503595

**Published:** 2026-04-10

**Authors:** Xiaolian Zhao, Jiaxin Yu, Nannan Jia, Chuzhang Hong, Yue Luo, Wantong Jing, Zhiming Feng, Xinhua Liu, Rui Tan

**Affiliations:** ^1^ School of Resources Environment and Materials Guangxi University Nanning China; ^2^ Department of Chemical Engineering Swansea University Swansea UK; ^3^ Department of Mathematics University of Manchester Manchester UK; ^4^ Department of Chemical Engineering University of Manchester Manchester UK; ^5^ School of Transportation Science and Engineering Beihang University Beijing China

**Keywords:** energy density, energy storage, high‐concentration electrolyte, multi‐electron transfer, redox flow battery

## Abstract

The inherent intermittency of energy sources such as solar and wind power hinders the transition to renewable energy, necessitating advanced energy storage solutions. Enhancing energy density is crucial for lowering system costs and enabling large‐scale deployment. Redox flow batteries (RFBs) demonstrate significant potential for grid‐scale energy storage due to their scalable capacity, high safety, and long cycle life. However, the relatively low energy density of conventional RFBs has hindered their widespread adoption. This review summarizes recent research progress in high‐energy‐density flow batteries, focusing on key parameters and strategies for enhancing the energy density of aqueous RFBs (ARFBs). Three core strategies are discussed in detail: broadening the cell voltage window, constructing multi‐electron transfer systems, and developing high‐concentration electrolytes. To overcome the practical challenges (e.g., species crossover, material degradation) associated with implementing these high‐energy‐density strategies, this review also highlights the critical role of advanced membrane technology as a key enabling component. Finally, the review outlines prospects and challenges for high‐energy‐density flow batteries, emphasizing the need for further research on material stability, energy efficiency, and cost‐effectiveness. Through continued innovation and optimization, high‐energy‐density flow batteries are expected to become a mainstream technology for grid storage, providing robust support for the efficient utilization of renewable energy.

## 1 Introduction

With societal advancement and population growth, conventional energy sources are becoming increasingly inadequate to meet human demands [1]. Growing concerns over the sustainability of fossil fuels and their environmental impact are accelerating the transition toward renewable energy [2, 3, 4, 5, 6]. The transformation of the global energy structure and the rapid development of renewable energy sources, such as solar and wind power, have gained significant momentum [7, 8, 9]. However, despite this strong momentum, the inherent intermittency and variability of renewables continue to pose severe challenges to the secure and stable operation of power grids [10, 11]. Compared to mechanical energy storage methods such as pumped hydro and flywheel energy storage, electrochemical energy storage offers advantages of flexible deployment and high efficiency [12, 13, 14, 15, 16]. Energy storage systems can mitigate power fluctuations from renewables, thereby ensuring secure and stable grid integration [17, 18, 19, 20, 21, 22, 23, 24]. Moreover, they can regulate grid input/output to facilitate the development of smart grids.

**FIGURE 1:**
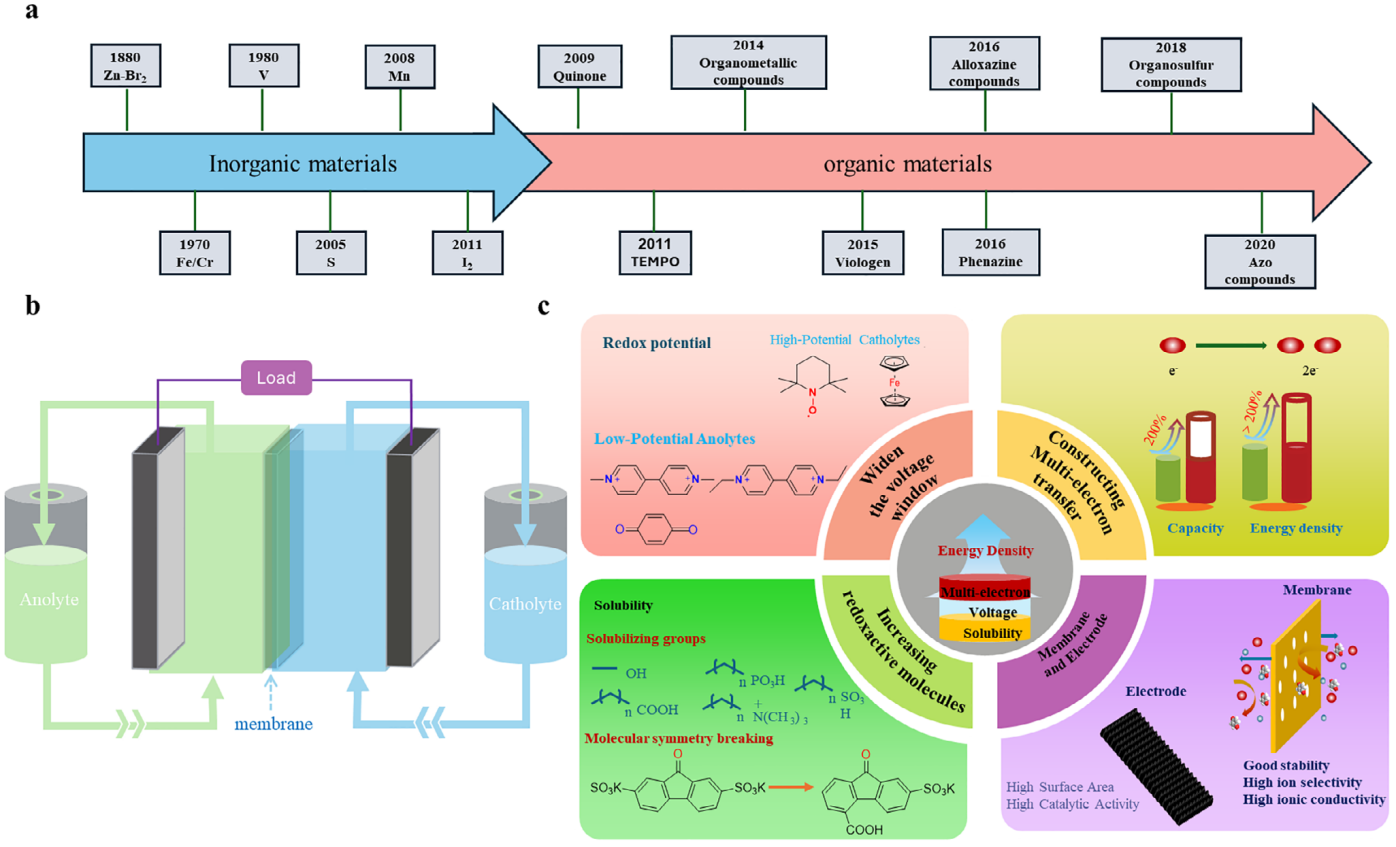
(a) Development of flow battery technologies. (b) Schematic illustration of redox flow battery. (c) Three core strategies and key components for enhancing the energy density of flow batteries.

In recent years, flow battery technology has advanced rapidly, evolving from early all‐iron and zinc–bromine systems to the currently dominant vanadium flow battery (VFB), with significant improvements in stability and cycle life (Figure 1a) [25, 26, 27, 28]. As an electrochemical energy storage technology, flow batteries store and release energy through redox reactions of active species in the electrolyte. Their unique architecture decouples energy storage units (electrolyte tanks) from power delivery units (stacks) (Figure 1b). This separation enables independent scaling of energy and power capacities, providing distinct advantages for grid peak‐shaving, renewable energy integration, and distributed energy systems [29, 30, 31, 32]. However, although traditional flow battery systems, particularly VFBs, are relatively mature technologies, their energy density remains constrained by fundamental limitations, typically limited to volumetric energy densities of only 25–50 Wh L^−1^ [33]. Moreover, the reliance on expensive perfluorosulfonic acid (PFSA) cation‐exchange membranes further increases the overall system cost of VFBs [34]. The U.S. Department of Energy projects that the levelized cost of storage (LCOS) for VFBs will reach 100–150 USD kW h^−^^1^ by 2030, restricting their application in space‐constrained scenarios and undermines overall cost‐effectiveness [35]. Compared to lithium‐ion batteries (250–700 Wh L^−^^1^) and sodium–sulfur batteries (150–300 Wh L^−^^1^), traditional flow batteries exhibit a significant competitive disadvantage in energy density [36]. Consequently, developing high‐energy‐density flow battery systems has emerged as a critical research priority in energy storage technology development [37, 38].

The theoretical energy density of RFBs is expressed by Equation (1) [39]:

*W* = *nCFV*/*µ*_*v* (1)

In Equation (1), *W* is the energy density (Wh L^−1^); *n* is the number of electrons transferred in the redox reaction; *C* is the minimum concentration of electroactive species dissolved in the electrolyte (mol L^−^^1^); *F* is the Faraday constant; *V* is the cell voltage (V); *µ*_v_ is the volume factor, defined as *µ*_v_ = 1 + (concentration of less soluble active species/concentration of more soluble active species). When concentrations are equal, *µ*_v_ = 2.

Based on this principle, strategies for enhancing the energy density of flow batteries can be classified into three main aspects: (1) widening the operational voltage window through the selection of suitable electrode active materials or optimization of the electrolyte environment; (2) achieving multi‐electron transfer processes by designing active materials capable of undergoing multiple redox reactions; (3) increasing the concentration of active materials by improving solubility or adopting novel electrolyte systems (Figure 1c). While these three strategies directly target the parameters in the energy density equation (Figure 1c), their practical implementation at high voltages and concentrations introduces significant challenges, including exacerbated active species crossover and accelerated membrane degradation. These challenges underscore that the viability of the three core strategies is intrinsically linked to membrane performance. Therefore, the development of high‐performance membranes should be regarded not as a standalone fourth strategy for boosting energy density, but rather as a critical enabling technology that determines the ultimate efficiency and longevity of high‐energy‐density flow batteries. Accordingly, this review dedicates a section to discussing recent progress in membrane materials as an indispensable supporting component. In recent years, researchers have conducted extensive and significant research efforts, leading to the emergence of numerous novel materials and technological pathways [40, 41]. This review systematically summarizes the current major strategies for improving the energy density of flow batteries, including voltage window expansion, multi‐electron transfer systems, and chemically regulated high‐concentration electrolyte solutions.

## 2 Expanding the Voltage Window: Enhancing Cell Potential Difference

**FIGURE 2:**
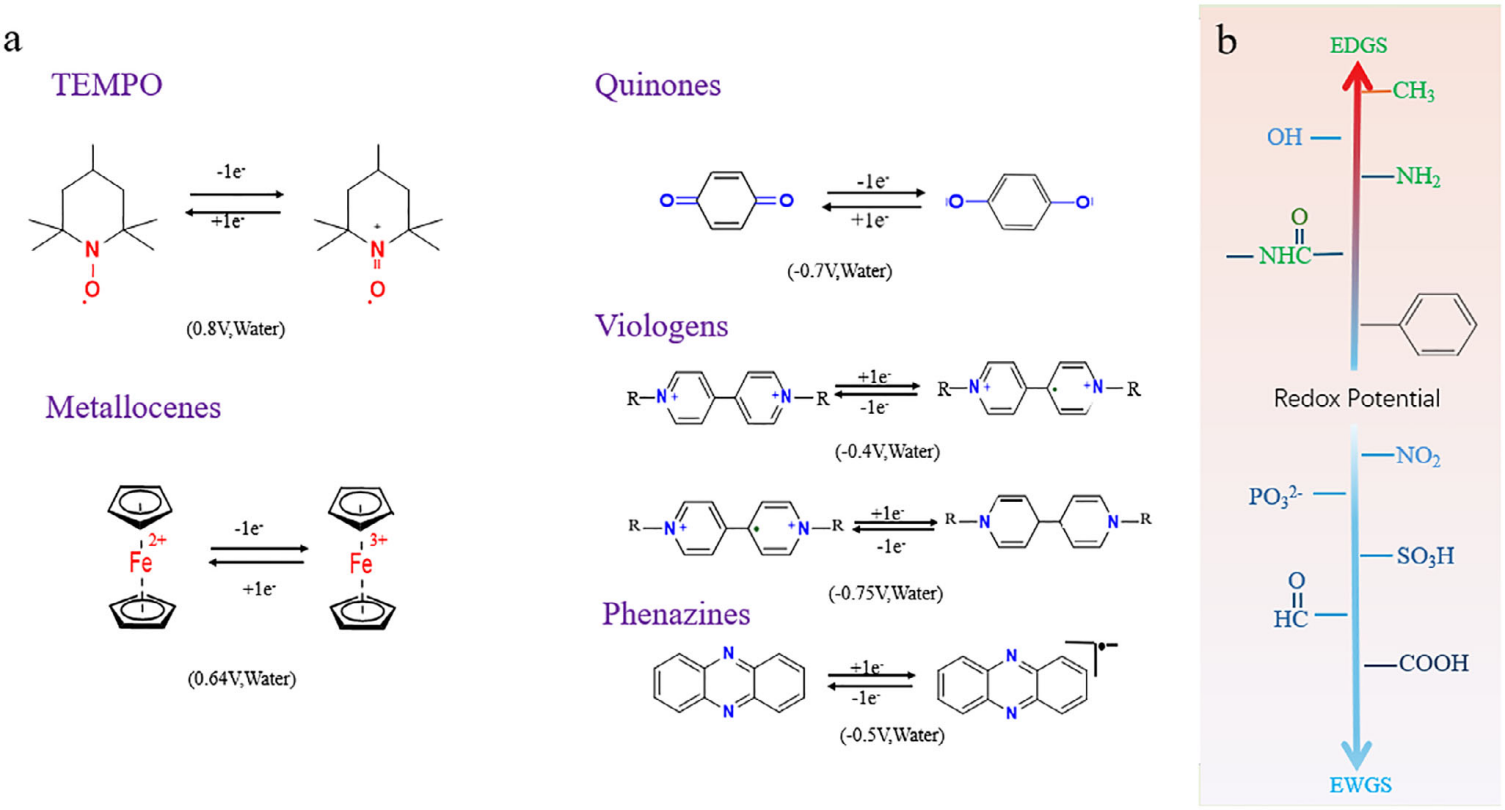
(a) The main classes of the organic compounds used in RFBs and standard ranges of the redox potentials vs. SHE. (b) Representative electron‐donating (activating) and electron‐withdrawing (deactivating) groups.

The energy density of flow batteries is predominantly determined by their operating voltage, which is directly governed by the difference in redox potentials between the positive and negative electrode active materials [42]. The first ionization energy of a molecule can be approximated by the negative value of its highest occupied molecular orbital (HOMO) energy, while the electron affinity corresponds to the negative value of its lowest unoccupied molecular orbital (LUMO) energy [43, 44]. Consequently, the oxidation potential is primarily dictated by the HOMO energy level, and the reduction potential by the LUMO energy level. There are numerous approaches for achieving high‐voltage flow batteries, including the development of novel electrolytes (e.g., “water‐in‐salt” electrolytes) to broaden the electrochemical stability window, as well as the design of high‐potential catholyte and low‐potential anolyte materials [45]. Significant progress has been achieved with both inorganic and organic active material systems. Among these, organic redox‐active molecules, owing to their unparalleled molecular tunability, provide an ideal platform for systematically elucidating structure–property relationships and investigating the physicochemical origins of potential modulation (Figure 2a). This section therefore, focuses on enhancing cell voltage through rational organic molecule design, aiming to establish universal design principles. Electron‐withdrawing groups (e.g., ─CN, ─NO_2_, ─CF_3_) lower both the HOMO and LUMO energy levels through synergistic *σ*‐ and π‐electron withdrawal effects, resulting in a positive shift in redox potentials [46]. Consequently, they are well‐suited for use as high‐potential cathode materials. In contrast, electron‐donating groups (e.g., ─NH_2_, ─OCH_3_, alkyl substituents) elevate these orbital energies via electron donation, inducing a negative potential shift that meets the requirements for low‐potential anolyte materials (Figure 2b) [47]. Owing to its predictability and precision in regulating electron density, this substituent effect strategy provides a powerful approach for the rational design of redox‐active molecules. A complementary strategy involves extending the π‐conjugation framework. Enhanced π‐electron delocalization typically elevates the HOMO energy while concurrently lowering the LUMO energy, thereby narrowing the HOMO–LUMO gap and facilitating thermodynamically favorable shifts in redox potentials [48]. For instance, incorporating conjugated bridging units, such as benzene [49, 50], thiophene [51], or selenophene [52, 53], into viologen derivatives can reduce the LUMO energy by 0.3–0.5 eV, corresponding to a commensurate negative shift in reduction potential [54]. Similarly, fusing additional benzene rings into anthraquinone (AQ)‐based systems progressively decreases the LUMO energy, enabling continuous and precise tuning of the reduction potential. This conjugation‐extension strategy significantly broadens the accessible range of potential regulation and enhances charge‐transfer properties, proving particularly advantageous for tailoring systems that demand substantial yet precise modulation of redox couples [55]. In the following section, we summarize the recent developments in high‐potential organic catholytes and low‐potential organic anolytes in the following section.

### 2.1 High‐Potential Catholyte Design

The development of high‐performance organic catholytes relies critically on precise molecular‐level control. Two representative classes are nitroxyl radicals, such as TEMPO and organometallic compounds, such as ferrocene. The molecular design of TEMPO derivatives is dedicated to 4‐position functionalization, aiming to synergistically mitigate stability issues such as crossover and degradation. In contrast, studies on ferrocene utilize substituent engineering of its cyclopentadienyl ligands to finely tune oxidation potential.

#### 2.1.1 TEMPO‐Based Catholytes

**FIGURE 3:**
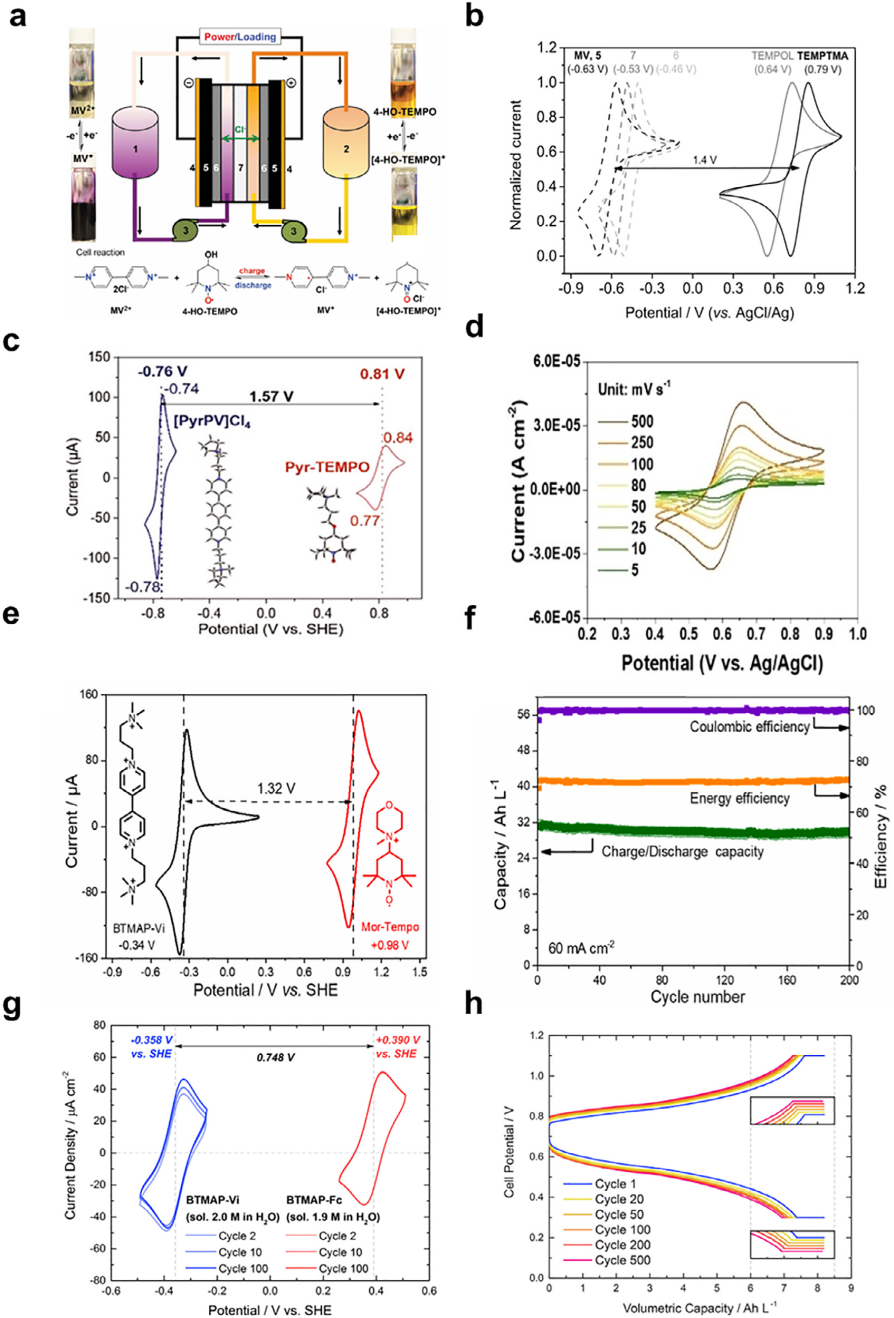
(a) Scheme of 4‐OH‐TEMPO‐MV flow battery. Reproduced with permission [58]. Copyright 2016, WILEY. (b) Cyclic voltammograms of TEMPO and viologen derivatives. Reproduced with permission [59]. Copyright 2016, WILEY. (c) Pyr‐TEMPO/[PyrPV]Cl. (d) Cyclic voltammograms (CVs) of MIAcNH‐TEMPO. (e) Cyclic voltammograms (CVs) of Mor‐TEMPO and BTMAP‐Vi. (f) Cycling performance of the Mor‐TEMPO/BTMAP‐Vi neutral aqueous redox flow battery. Reproduced with permission [57]. Copyright 2025, Elsevier. (g) Cyclic voltammograms of BTMAP‐Vi and BTMAP‐Fc. (h) Galvanostatic charge/discharge voltage profiles. Reproduced with permission [65]. Copyright 2017, American Chemical Society.

Nitroxyl radicals, such as 2,2,6,6‐tetramethylpiperidine‐1‐oxyl (TEMPO) and its derivatives, have garnered widespread interest due to their reversible N─O/N = O^+^ redox chemistry [56, 57]. From a molecular orbital perspective, their HOMO is predominantly localized on the nitroxyl moiety, with its energy directly governing the oxidation potential. Liu [58] first reported the hydroxyl‐functionalization of TEMPO (4‐OH‐TEMPO) and its application in all‐organic redox flow batteries (AORFBs), achieving a high oxidation potential of +0.8 V vs. SHE. When paired with methyl viologen (MV), the system delivered a cell voltage of 1.25 V while maintaining near 100% Coulombic efficiency even at 100 mA cm^−^^2^ (Figure 3a). However, 4‐OH‐TEMPO lacks charged functional groups, making it susceptible to membrane crossover due to insufficient electrostatic repulsion. Its small molecular size further exacerbates permeation issues. Moreover, the 4‐position hydroxyl group in the oxidized state is prone to over‐oxidation, generating a carbonyl moiety that reduces aqueous solubility and accelerates degradation. In a 0.5 M 4‐OH‐TEMPO||MV system, the capacity retention drops to 89% after 100 cycles, corresponding to a decay rate of 0.11% per cycle. These stability challenges have become the driving force for subsequent molecular engineering efforts.

To mitigate the crossover and degradation of 4‐OH‐TEMPO, researchers introduced electron‐withdrawing, charged groups at the 4‐position. This dual‐functional modification simultaneously enhanced the redox potential and suppressed crossover. Janoschka et al. [59] developed a quaternary ammonium‐functionalized TEMPO derivative (TEMPTMA), where the electron‐withdrawing effect elevated its oxidation potential by 0.15 V (to +1.0 V vs. SHE) compared to 4‐OH‐TEMPO. In the MV||TEMPTMA system, the catholyte achieved a volumetric capacity of 54 Ah L^−1^ and an energy density of 38 Wh L^−1^. This strategy exemplifies precise molecular‐level control, although it requires a trade–off between synthetic complexity and the magnitude of potential enhancement. Building on this foundation, Liu and Aziz's teams proposed a structure‐refinement strategy to further optimize TEMPO derivatives. The TMAP–TEMPO derivative, reported by Liu and Aziz et al. [60] features a long‐side‐chain quaternary ammonium group tethered via an ether linkage. Although the steric shielding effect reduced its redox potential to +0.81 V (vs. SHE), the extended chain dramatically suppressed both self‐discharge reactions and membrane crossover, thereby enhancing cycling stability. Hu et al. [61] synergized the long‐side‐chain advantage of TMAP‐TEMPO with the high‐potential feature of TEMPTMA, designing the N_2_‐TEMPO derivative. This molecule incorporates two quaternary ammonium groups directly linked to the TEMPO core and extended branched chains, raising its redox potential to +1.0 V (vs. SHE). When paired with BTMAP‐Vi, the system delivered a cell voltage of 1.35 V, achieving dual optimization of potential and stability. Currently, Pyr‐TEMPO, a pyrrolidinium‐functionalized derivative, was developed. Coupled with [PyrPV]Cl_4_, RFB exhibited an open‐circuit voltage of 1.57 V, significantly enhancing cycle durability while boosting energy density to 16.8 Wh L^−1^ (Figure 3c) [62]. Incorporating sterically hindered conjugated functional groups enables precise modulation of nitroxyl radical molecular conFigureurations, thereby significantly enhancing both electrochemical stability and the energy density of corresponding battery systems. The synthesis of pyrrolium‐modified TEMPO (MIAcNH‐TEMPO) and its pairing with Zn in an aqueous zinc‐hybrid flow battery (AZHFB) achieved an exceptionally high open‐circuit voltage (OCV) of 1.71 V, surpassing most existing TEMPO‐based systems (Figure 3d) [57, 63]. The incorporation of cyclic amine groups leverages their strong electron‐withdrawing effects to reduce charge density at the nitroxyl radical's active site. This modification yielded Mor‐TEMPO, with a high redox potential of 0.95 V (vs. SHE). When deployed in RFB, the system exhibited enhanced energy density (Figure 3e, f).

#### 2.1.2 Ferrocene‐Based Catholytes

In contrast, the redox process of ferrocene involves the transition between d^6^/d^5^ electronic configurations at the iron center. Its HOMO primarily consists of an antibonding combination of Fe 3d orbitals and Cp ring π orbitals, while the LUMO corresponds to the antibonding orbital of Fe 3d‐Cp π [64]. The standard oxidation potential of unsubstituted ferrocene is +0.40 V (vs. SHE). Through the introduction of substituents with different electronic effects, this potential can be tuned within the range of +0.1 V to +0.8 V. According to the linear free energy relationship, the formal potential of ferrocene derivatives exhibits an excellent linear correlation with the substituent constant: E° = 0.40 +0.12 Σσ. Hu et al. [39]. reported the introduction of a trimethylammonium halide group into FcNCl, achieving a redox potential of +0.61 V (vs. SHE). This modified compound was assembled with MV to construct an aqueous organic flow battery. Utilizing a low‐cost AMV membrane, the system demonstrated a high theoretical energy density of 45.5 Wh L^−1^ and exhibited excellent cycling performance across current densities ranging from 40 to 100 mA cm^−^^2^. Kim et al. further combined bromine atoms with quaternary ammonium groups, attaining a higher potential of +0.675 V. While this strategy offers advantages such as low cost and predictable tuning, the extent of potential modulation remains limited due to constraints in orbital hybridization characteristics. In a separate approach, Li's group employed β‐cyclodextrin to encapsulate insoluble ferrocene derivatives, stabilizing the potential within the range of +0.50‐0.53 V and mitigating stability degradation issues. Eugene et al. [65] developed a double‐long‐chain quaternized BTMAP‐Fc (Figure 3g, h), which not only shifted the potential positively to +0.39 V but also enhanced electron transport through extended conjugation. This design is suitable for membrane‐free conFigureurations, thereby reducing costs. They also systematically investigated the influence of trimethylammonium groups with varying branch chain lengths on the electrochemical properties of ferrocene. Molecular orbital calculations revealed an inverse relationship between the redox potential of ferrocene‐based active materials and the LUMO energy level. Among them, BQH–Fc, with the lowest LUMO electron density, exhibited the best cycling stability, providing critical guidance for the rational design of high‐stability ferrocene‐based electrolytes in future studies.

Beyond established systems like TEMPO and ferrocene, researchers are actively exploring high‐potential cathode materials based on novel chemical frameworks. While these designs similarly adhere to the principle of molecular orbital engineering, they emphasize the selection of distinct mechanisms to meet specific application requirements.

Organic sulfur compounds, such as disulfides, undergo redox reactions involving the cleavage and formation of S─S bonds. Their HOMO is primarily contributed by the lone pair electrons on sulfur atoms, while the LUMO corresponds to the σ* orbital of the S─S bond. The introduction of electron‐withdrawing substituents can lower both the HOMO and LUMO energy levels, thereby modulating both the redox potentials, making these compounds suitable for high‐voltage applications. Furthermore, the extension of π‐conjugation narrows the bandgap, enhancing reaction kinetics. Heterocyclic compounds like carbazole and phenothiazine possess extended π‐conjugated systems and benefit from heteroatom effects. Their HOMO, formed by the conjugation of the heteroatom's lone pair with the π‐system, initially resides at a relatively high energy level. By incorporating electron‐withdrawing groups to lower HOMO energy, the oxidation potential can be elevated beyond +0.8 V (vs. SHE). The key advantage of this strategy lies in its wide tunability, making it particularly suitable for scenarios demanding high stability. Metal complexes, such as iron‐bipyridine systems, feature electronic structures characterized by interactions between metal d‐orbitals and ligand orbitals. The redox potential can be precisely tuned within a range of approximately +0.4 to +1.1 V through strategic ligand substitution. The synergistic extension of π‐conjugation within the ligands enhances coordination stability and suppresses side reactions.

### 2.2 Low‐Potential Anolyte Design

#### 2.2.1 Anolytes Based on Viologen Derivatives

Viologen derivatives stand out as one of the most promising candidate materials due to their unique molecular structure and electrochemical properties [66]. Compared to other organic anode materials, the rigid planar structure of viologen based on the 4,4'‐bipyridine skeleton endows it with excellent electrochemical stability and reversibility. It also exhibits outstanding aqueous solubility, reaching 2–3 M, which is significantly higher than most organic electroactive molecules and directly determines the upper limit of the battery's energy density [67]. The development of high‐performance viologen‐based flow batteries is challenged by the inherently high redox potential of viologens (e.g., −0.54 V for BTMAP‐Vi vs. Ag/AgCl), a consequence of the strong electron‐withdrawing nature of the pyridinium ring. Therefore, designing novel viologen molecules with lower potential, high reversibility, and superior stability is a pivotal challenge [68].

The reduction potential of the basic MV^2^^+^ is approximately −0.45 V (vs. SHE), providing a favorable electrochemical window for constructing high‐voltage batteries. Liu et al. [69] first paired MV with 4‐hydroxy‐2,2,6,6‐tetramethylpiperidin‐1‐oxyl (4‐OH‐TEMPO) to construct an aqueous organic redox flow battery (AORFB) achieving a notably high cell voltage of 1.25 V. However, MV undergoes dimerization during the reduction process, resulting in a rapid capacity decay rate of up to 27.5% per day. Xiang et al. [70] further revealed that the radical cation MV^+^·tends to form molecular aggregates in aqueous solution to reduce its reactivity and overall system energy. This aggregation behavior also influences the energy level of its HOMO, leading to a positive shift in reduction potential with increasing concentration, which further constrains its practical applicability.

**FIGURE 4:**
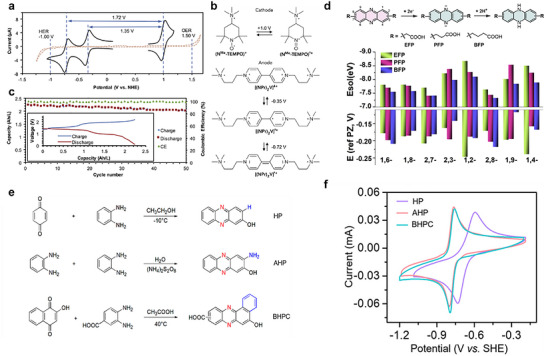
(a) Cyclic voltammograms of 4.0 mM [(NPr)_2 _V]Cl_4_ (−0.35 and −0.72 V) and 4 mM N^Me^‐TEMPO (1.0 V) in 0.5 M NaCl aqueous electrolyte. (b) Battery reactions of the [(NPr)_2 _V]Cl_4_/N^Me^‐TEMPO AORFB. Reproduced with permission [71]. Copyright 2017, Elsevier. (c) Extended 50‐cycle data of the [(NPr)_2 _V]Cl_4_/N^Me^‐TEMPO AORFB show discharge capacity and Coulombic efficiency vs. cycle number. Reproduced with permission [72]. Copyright 2020, WILEY. (d) Density functional theory (DFT)‐calculated reduction potentials and solvation energies for EFPs, PFPs, and BFPs. Reproduced with permission [85]. Copyright 2021, Elsevier. (e) Synthesis routes of HP, AHP, and BHPC molecules. (f) CV curves of HP, AHP, and BHPC molecules. Reproduced with permission [87]. Copyright 2020, American Chemical Society.

To address the stability issues and modulate the reduction potential of MV, DeBruler et al. [71] proposed the introduction of a quaternary ammonium group into the MV molecular structure, designing (Me)(NPr)V with a permanent +3 charge. The electrostatic repulsion between the introduced positive charges effectively suppresses the dimerization tendency of the radical species. This molecule exhibits two‐electron storage capability, with the second reduction potential negatively shifted by 0.39 V compared to the first step, yielding an average reduction potential of −0.6 V (vs. SHE), which significantly enhances the battery's operating voltage (Figure 4a, b). An AORFB assembled using this molecule as the anode achieved a theoretical energy density as high as 79.5 Wh L^−^^1^ (Figure 4c), setting a record at the time. Building upon this foundation, Jin et al. [72] developed a phosphonate‐functionalized viologen, 1,1'‐bis(3‐phosphonopropyl)‐[4,4'‐bipyridine]‐1,1'‐diium (BPP‐V), for use as an anode material. The strong coordination ability and anionic characteristics of the phosphonate groups not only effectively prevent the crossover of active species through the membrane but also maintain good electrochemical activity, offering a novel strategy to address the issue of cross‐contamination in flow batteries. With a reduction potential of ‐0.462 V (vs. SHE), BPP‐V was paired with a potassium ferrocyanide cathode, achieving an open‐circuit voltage of 0.9 V under pH 9 conditions.

Bridge group selection depends on π‐electron‐donating strength. Selenophene most strongly raises the LUMO level due to selenium's large atomic radius and strong π‐donation, thiophene shows a moderate effect, and benzene provides a smaller but clear negative shift [73, 74]. This concept was first validated by Luo et al. [75], who designed a π‐conjugation‐extended viologen derivative, [(NPr)_2_TTz]Cl_4_, featuring a planar thiazolo[5,4‐d]thiazole bridge. The extensive π‐conjugated system moderates the electron‐withdrawing interaction between the pyridinium units while preserving the excellent electrochemical activity characteristic of viologens through π‐extension. This electronic modulation resulted in a small potential separation of only 0.116 V between the two single‐electron reduction steps. However, the electron‐deficient nature of the thiazole bridge limited further negative potential shifts. Inspired by this work, Zhang et al. [51] employed a selenophene bridge to synthesize selenophene‐bridged viologen (SeV), achieving a significantly negative second reduction potential of −0.81 V vs. SHE. Concurrently, Pan et al. [49] reported a benzene‐bridged viologen derivative, [PyrPV]Cl_4_, which exhibited a reduction potential of −0.76 V (vs. SHE). Similarly, the research group of S. and Liang Z.X. [50] designed (APBPy)Cl_4_ using a benzene bridge, which also yielded a comparable negative shift and enhanced radical state stability.

However, the π‐conjugated extension strategy introduces new challenges while reducing the potential. The enlargement of the conjugated system increases molecular hydrophobicity, thereby decreasing its water solubility. Therefore, appropriate hydrophilic groups must be incorporated into the molecular design to counteract this effect.

#### 2.2.2 Phenazine‐Based Anolyte Materials

Phenazine and its derivatives have emerged as promising anode materials for aqueous organic flow batteries, attracting significant attention due to their unique two‐electron storage capability and highly tunable redox potentials [76, 77, 78]. The reduction potential of phenazine can be systematically modulated by introducing electron‐donating groups with varying strengths and at different positions [79, 80].

Hydroxy (─OH) and sulfonic acid (─SO_3_H) groups are among the most widely used and effective electron‐donating and hydrophilic modifiers [81, 82]. Hollas et al. designed and synthesized 7,8‐dihydroxyphenazine‐2‐sulfonic acid (DHPS). By incorporating both strongly electron‐donating hydroxy and sulfonic acid groups in an asymmetric conFigureuration, the molecule exhibited a significantly shifted reduction potential of −0.86 V (vs. SHE), which was one of the lowest reported values for phenazine derivatives at the time. When paired with a Fe(CN)₆^3^^−^/^4^^−^ cathode, the full cell achieved a voltage of 1.4 V. It demonstrated stable performance over 500 cycles at 100 mA cm^−^^2^ with energy efficiency exceeding 75%, underscoring the effectiveness of molecular design in tuning potential to enhance battery voltage.

Besides hydroxy and sulfonic acid groups, amino (─NH_2_) and alkoxy (─OR) groups also serve as effective electron‐donating substituents. Studies indicate that the strength, number, and positional arrangement of these groups collectively determine the extent of potential modulation [83, 84]. As shown in Figure 4d, in most cases, shorter alkyl chains provide lower reduction potentials. Generally, stronger and more numerous electron‐donating groups lead to greater elevation of the HOMO energy level, resulting in more negative reduction potentials [85]. For instance, introducing such groups at the 5,10‐positions (para to the pyrazine nitrogens) of the phenazine core facilitates more effective electron delocalization and a larger negative shift in potential [86].

Besides adding functional groups, extending the π‐conjugated system also lowers the reduction potential. Wang et al. [87] developed a series of fused‐ring phenazine derivatives, including benzo[a]hydroxyphenazine‐7,8‐carboxylic acid (BHPC), by annulating benzene rings onto the phenazine core (Figure 4e, f). This fusion strategy not only enhanced molecular planarity and conjugation, with the promoted electron delocalization and the further reduced reduction potential (e.g., BHPC reached −0.78 V vs. SHE) while improving electrochemical stability.

Rational molecular design enables precise control over the redox potential of electrode materials. Strategies such as introducing electron‐donating or electron‐withdrawing groups and expanding π‐conjugated systems enhance the operating voltage of flow batteries, thereby increasing their energy density. Systematic tuning of molecular orbital energy levels in cathode materials (e.g., TEMPO, ferrocene derivatives) and anode materials (e.g., AQ, quinoline derivatives) has driven significant advancements in high‐voltage aqueous organic flow batteries. However, the intrinsic electrochemical window of aqueous electrolytes (1.23 V) and material stability at extreme potentials limit further improvements [88]. Therefore, broadening the voltage window alone cannot satisfy the demand for high energy density. Developing active materials with multi‐electron transfer capabilities has become another key approach to enhancing theoretical capacity and energy density. Materials like AQ, phenazine, and AQ possess inherent multi‐electron reaction characteristics while enabling potential regulation. For instance, quinoline derivatives and π‐extended purpurocyanidins undergo reversible two‐electron reactions, synergistically enhancing both operating voltage and electron storage capacity—merging the “high‐voltage” and “multi‐electron” strategies. However, despite achieving partial multi‐electron transfer, systematic investigations into the reaction mechanisms, intermediate stability, electron transfer dynamics, and actual energy storage capabilities of these materials remain essential.

## 3 Multi‐Electron Transfer Systems

Designing single‐molecule materials for multi‐electron redox processes provides a key alternative to voltage window expansion for increasing flow battery capacity [89, 90]. The number of electrons transferred per molecule *n* is directly proportional to the theoretical capacity (*C*  =  *nF*/*V*). Multi‐electron reactions at a single molecular scaffold thus improve energy storage density without raising electrolyte volume or viscosity. This approach helps reduce system costs and space requirements.

The key to achieving efficient multi‐electron transfer lies in modulating the thermodynamic and kinetic balance of each electron transfer step through molecular design, such that the reaction potentials are brought as close as possible [91, 92]. This enables a flat plateau in the discharge curve and avoids capacity and energy efficiency losses caused by excessive potential differences [93]. Theoretically, multi‐electron processes follow a sequential potential model: an n‐electron reaction is decomposed into multiple single‐electron steps. The potential differences between steps are jointly determined by molecular structure, solvation effects, and intramolecular electrostatic interactions. Kinetically, two pathways exist: cooperative (electron‐coupled) and stepwise (electron‐decoupled) transfer. Cooperative transfer relies on strong electron interactions to achieve synergistic mechanisms; stepwise transfer requires similar standard rate constants for each step to prevent a single slow step from limiting overall kinetics. For FBs, moderate electron coupling typically optimally balances reversibility and reaction rate. Designing stable intermediates can also suppress disproportionation reactions, extending cycle life. This strategy has shown promise in multivalent systems involving halogens and transition metals in recent years, though the stability of high‐valent intermediates and insufficient reaction rates remain key challenges. The following sections examine how these fundamental pathways, namely cooperative and stepwise, manifest in different material classes, from inorganic halogens and transition metals to tunable organic and polymeric systems, each presenting distinct design imperatives.

### 3.1 Halogen‐Based Systems

**FIGURE 5:**
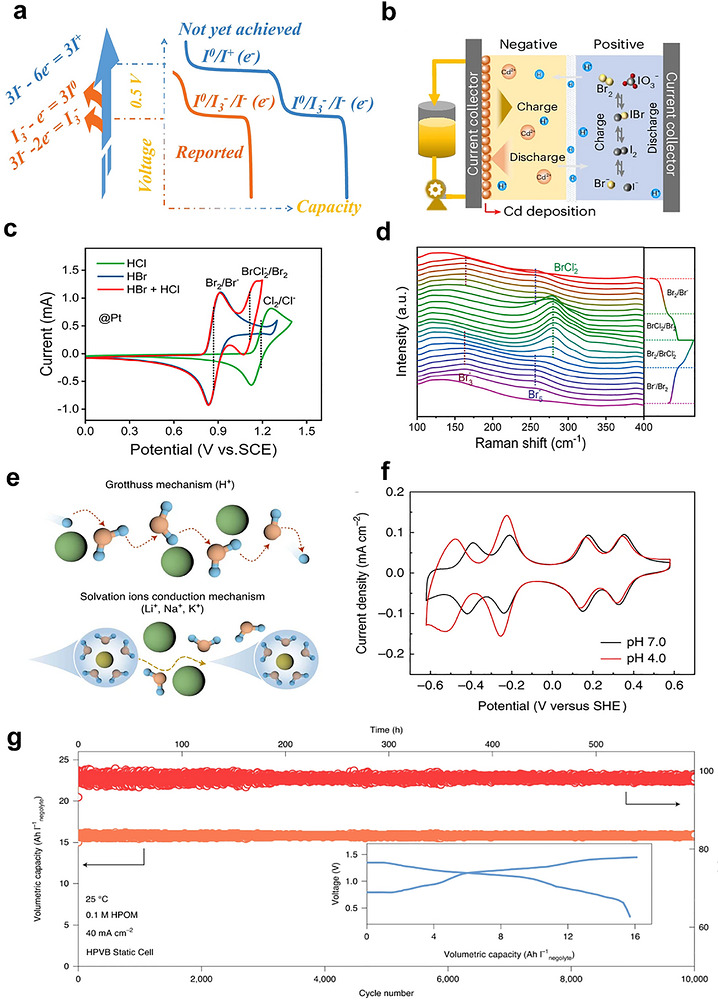
(a) The discharge curves of the iodine‐based FB. Reproduced with permission [99]. Copyright 2021, Royal Society of Chemistry. (b) Schematic of an assembled battery with a multi‐electron transfer cathode and cadmium metal as the anode. Reproduced with permission [100]. Copyright 2024, Springer Nature. (c) Cyclic voltammogram of HBr, HCl, and HBr + HCl. (d) Characterization of reaction mechanism of the catholyte. Reproduced with permission [101]. Copyright 2022, American Chemical Society. (e) Schematics of the Grotthuss mechanism of proton and solvated ions conduction mechanism. Reproduced with permission [110]. Copyright 2018, Springer Nature. (f) Cyclic voltammograms of Li_6_[P_2_W_18_O_62_]. (g) Cycling performance of the HPVB static cell using 0.1 M HPOM as catholyte and 1.5 M VOSO_4_ as anolyte at 40 mA cm^−2^. Reproduced with permission [108]. Copyright 2022, Springer Nature.

Halogen elements possess inherent advantages for multi‐electron transfer and enhanced energy density in flow batteries, owing to their multiple accessible oxidation states (−1, 0, +1, +5, +7) and highly reversible electrochemical interconversions [94, 95, 96]. Conventional redox couples, such as I_3_^−^/I^−^ and Br_3_^−^/Br^−^ [97], typically undergo one‐electron processes. However, recent research has successfully enabled reversible two‐electron, and even multi‐electron, transformations by strategically modulating the reaction pathway and stabilizing key intermediates [98]. Li et al. [99] successfully stabilized the I^+^ cation by introducing Cl^−^ anions into the electrolyte, thereby activating the second‐step redox couple of I^0^/I^+^. The underlying mechanism involves the formation of a stable ICl_2_^−^ complex between Cl^−^ and I^+^, which reduces the reactivity of I^+^ and enables its participation in reversible electrochemical reactions. This strategy facilitates a two‐electron transfer process from I^−^ to I^+^ in iodine‐based flow batteries, elevating the redox potential plateau by approximately 0.5 V and achieving an approximately 238% increase in energy density in dual‐plateau discharge mode (Figure 5a). Xie et al. [100] demonstrated a reversible I^−^/IO_3_^−^ redox pathway under strongly acidic conditions, wherein Br^−^ participates in the reaction by forming IBr and Br_2_ as critical intermediates. Employing a 6 M I^−^ catholyte and a Cd/Cd^2^^+^ anode, the assembled cell achieved an exceptional energy density exceeding 1200 Wh L^−^^1^ (based on the catholyte volume) (Figure 5b). Although iodine possesses higher oxidation states, such as +5 and +7 (e.g., in IO_3_^−^ and IO_4_^−^), which theoretically enable multi‐electron transfer reactions, their practical exploitation remains in the exploratory stage due to sluggish electrochemical kinetics and poor reversibility.

Bromine‐based flow batteries also rely on the Br_2_/Br^−^ single‐electron pair. Developing multi‐electron bromine chemistry presents challenges, primarily due to the tendency of highly oxidized bromine species (such as Br^+^ in HBrO) to undergo irreversible reactions. Xu et al. [101] leveraged the complexation effect between Cl^–^ and Br^+^ to construct a BrCl_2_^−^/Br^−^ two‐electron transfer system, achieving a high discharge capacity of up to 96 Ah L^−1^ in a Ti‐Br‐Cl flow battery (Figure 5c, d). Slow kinetics in the second electron transfer at high current densities lowered voltage efficiency. Studies also found Br^+^ intercalates into graphite, forming Br‐Graphite Intercalation Compounds (Br‐GIC). This compound offers a potential about 0.5 V higher than the Br_2_/Br^−^ couple and increases energy density by 65% [102]. The multi‐electron redox chemistry of halogens (e.g., iodine and bromine) is intrinsically stepwise in nature, stemming from the necessity to traverse distinct high‐valent intermediate states (e.g., I^+^, Br^+^) during oxidation state changes. These odd‐valent halogen species are highly reactive and susceptible to disproportionation or irreversible side reactions, posing a fundamental challenge for achieving reversible multi‐electron transfer. Therefore, the core research endeavor in this domain focuses on stabilizing these pivotal intermediates within the stepwise framework. Strategies such as anion coordination (e.g., formation of ICl_2_^−^ or BrCl_2_^−^ complexes) or incorporation into solid‐state matrices (e.g., Br‐graphite intercalation compounds, Br‐GICs) aim to create a stabilized local chemical environment for these high‐energy species. By doing so, the inherently sequential stepwise reactions are effectively “concatenated” into an apparently efficient and reversible multi‐electron process. This mechanistic insight fundamentally explains why performance advancements in halogen‐based systems critically depend on sophisticated electrolyte formulation and meticulous interface engineering. However, Br‐GIC stability needs improvement. Stabilizing high‐valent active species and coordinating their reaction kinetics remain the main challenges for halogen‐based multi‐electron systems.

### 3.2 Transition Metal‐Based Systems

Transition metals support multi‐electron transfer in flow batteries because they access multiple oxidation states. Manganese‐based flow battery research targets the multi‐electron reaction from Mn^2^^+^ to solid MnO_2_. This approach addresses the capacity limitation of the traditional single‐electron MnO_2_/Mn(OH)_2_ reaction. In manganese‐based systems, Ye et al. [103] established a two‐electron conversion pathway (Mn^2^^+^→Mn^3^^+^→Mn^4^^+^), effectively doubling the theoretical capacity. However, the intermediate Mn^3^^+^ is prone to disproportionation in solution, generating insoluble MnO_2_ and leading to capacity fade [104]. To address this, pioneering work demonstrated this concept via a constant‐voltage charging protocol (at 2.2 V) in a Mn–H_2_ static cell, electrodepositing γ‐MnO_2_ and confirming the solid–liquid conversion reversibility through in situ characterization. This system exhibited exceptional kinetics and stability, enduring over 10,000 cycles at 100 mA cm^−^^2^ without decay [105]. The scope was subsequently expanded to Zn–MnO_2_ conFigureurations, achieving remarkable energy densities up to 409 Wh kg^−1^ in static cells. To circumvent the impracticality of constant‐voltage charging for large‐scale applications, research has evolved toward electrolyte engineering. Mn^3^^+^, as a well‐defined thermodynamic intermediate, is an essential step in the overall reaction pathway. However, its inherent disproportionation tendency (2Mn^3+^→Mn^2^^+^+Mn^4^^+^) constitutes a major channel for capacity fade. Consequently, the fundamental rationale behind recent breakthroughs in electrolyte engineering, such as the introduction of weak Brønsted acids or ligands, lies in modulating proton transfer or coordination environments to alter the rate‐determining step or the lifetime of intermediates, thereby circumventing the unstable free Mn^3^^+^ ion state and enabling a more direct and efficient two‐electron solid–liquid conversion. Innovative approaches utilizing weak Brønsted acid additives [106] or acetate anions (e.g., in Mn(Ac)_2_ electrolytes [107]) have emerged. These strategies function as efficient proton donors or ligands, respectively, lowering the Mn^2^^+^/MnO_2_ conversion overpotential, facilitating MnO_2_ dissolution, and entirely suppressing Mn^3^^+^ formation. This has enabled reversible cycling in neutral media with Mn concentrations up to 1 M. The Zn^2^^+^/Zn redox couple enables a two‐electron deposition/stripping reaction with a high theoretical specific capacity of 820 mAh g^−1^, operating at potentials of −0.76 V and −1.26 V (vs. SHE) in neutral and alkaline media, respectively [108]. However, dendrite growth and inhomogeneous deposition remain the primary challenges for long‐term cycling. To address this, Wang et al. employed a truncated pyramid‐shaped carbon felt (TCF) to guide uniform zinc deposition, achieving an areal capacity of 240 mAh cm^−2^ [109].

### 3.3 Polyoxometalate‐Based Systems

Polymetallic oxyanions (POMs) are molecular clusters composed of heteroatoms and transition metal ions, characterized by structural diversity and outstanding electrochemical properties. A single POM molecule contains multiple metal redox centers, enabling reversible multi‐electron transfer. The electron transfer can be stepwise, with discrete redox waves for each center (e.g., the two‐step, four‐electron process of W^6+^/W^5+^), or approach cooperative behavior if the centers are electronically decoupled and operate at similar potentials. The delocalized electronic structure of its metal‐oxygen framework also ensures rapid electron transfer kinetics. The macromolecular size of POMs effectively limits their transmembrane permeation in ion‐exchange membranes, providing an inherent advantage for developing multi‐electron flow batteries. Theoretically, a single POM molecule can participate in the reversible transfer of more than 10 or even dozens of electrons.

In the early stages of development, Chen et al. [110] pioneered the investigation into the feasibility of vanadium‐substituted Keggin‐type polyoxometalates (POMs) for RFB applications, specifically demonstrating the performance of K_6_H[A‐α‐SiV_3_W_9_O_40_]. Their seminal work highlighted a critical structure‐property relationship: the Keggin‐type architecture, characterized by its relatively lower charge density, offers superior structural stability compared to the Wells‐Dawson‐type structure, making it a more promising candidate for long‐term electrochemical cycling. Liu et al. developed a fully symmetric system based on H_6_[CoW_12_O_40_], wherein both the cathode and anode employ the two‐step, four‐electron transfer process of the tungsten centers (W^6^^+^/W^5^^+^). Although the total number of transferred electrons per molecule was limited, resulting in a practical energy density of 15.4 Wh L^−^^1^, this work successfully demonstrated the fundamental feasibility and operational concept of an all‐symmetric, multi‐electron redox system. To further enhance electron utilization, Yang et al. [111] employed the [P_2_W_18_O_62_]^6−^ polyanion, achieving a remarkable 18‐electron transfer process, which corresponded to a high volumetric energy density of 225 Wh L^−^^1^. However, the practical deployment of this system is constrained by its moderate solubility of 0.5 M (Figure 5e), posing a significant challenge to achieving higher areal capacity and energy density in practical devices.

The low solubility of POMs limits their application in energy storage. To address this challenge, two promising strategies have been identified: (1) the rational selection of counter‐cations (e.g., replacing K^+^ with Li^+^ to leverage the higher lattice energy and smaller hydrated radius) and (2) the strategic choice of POM structural types (e.g., Keggin vs. Wells–Dawson), which can significantly optimize both solubility and electrochemical stability, thereby enhancing overall device performance. Ai et al. [108] demonstrated that employing protons as the counter‐cations dramatically enhanced the solubility of H_6_P_2_W_18_O_62_ (denoted as HPOM). A resulting proton‐mediated HPOM‐based flow battery (HPVB), conFigureured with a VOSO_4_ anode and an HPOM cathode, exhibited exceptional low‐temperature resilience, maintaining stable operation at −20°C for over 1200 h and achieving a peak power density of 282.4 mW cm^−^^2^ (Figure 5f, g). Concurrently, the use of POMs such as Li_5_BW_12_O_40_, which undergo reversible two‐electron redox reactions under mild conditions (pH 3–8), effectively circumvents the corrosion problems associated with strong acidic electrolytes. Batteries incorporating these materials demonstrated outstanding cycling stability, retaining 95% of their capacity over 300 cycles alongside a high average Coulombic efficiency of 99.79%.

### 3.4 Molecular Multi‐Electron Systems

Organic molecules offer more promise than inorganic systems for multi‐electron flow batteries because of their structural diversity and design flexibility. Molecular engineering enables these materials to store and release multiple electrons in redox reactions. The most investigated systems include viologens, quinones, and nitrogen‐containing heteroaromatic compounds, particularly those undergoing two‐electron transfers.

As introduced in Section 2.2.1, viologen derivatives are prominent low‐potential anolyte materials that also exhibit two‐electron storage capability. Mechanistically, their reduction follows a classic stepwise transfer pathway via two sequential one‐electron steps (Vi^2+^→Vi^+^→Vi^0^). While the first electron transfer is highly reversible, the second suffers from poor reversibility due to structural reorganization and dimerization of the neutral Vi^0^ species. This creates a significant potential separation between the two redox steps (typically >0.3 V), limiting voltage efficiency and cycle life. Molecular engineering strategies, such as incorporating permanent charges or extending π‐conjugation, have been developed to stabilize the reduced states and reduce this potential separation. These modifications help transform the stepwise process into a more cooperative‐like behavior, thereby enhancing two‐electron reversibility. Viologens thus exemplify how understanding the underlying electron transfer mechanism is essential for guiding molecular design toward high‐performance multi‐electron storage.

Unlike viologen systems where the core challenge involves regulating potential differences, quinones exhibit inherent chemical instability during redox processes. Quinone molecules remain susceptible to nucleophilic attack or disproportionation reactions at various oxidation levels. Their two‐electron reduction typically follows a stepwise pathway, involving a semiquinone radical anion intermediate. The closeness of the two redox potentials is a key metric for evaluating the efficiency of the overall process. The two carbonyl groups (C═O) in quinone compounds natively endow them with the capability for two‐electron redox reactions. Research efforts have therefore focused on molecular modifications to enhance their solubility and chemical stability, aiming to prevent side reactions such as Michael additions and disproportionation, thereby enabling the full utilization of their two‐electron capacity. In the case of benzoquinone (BQ) derivatives, early compounds like BQDS (1,2‐benzoquinone‐3,5‐disulfonic acid) suffered from poor stability [112]. This was primarily due to the susceptibility of their unsubstituted active sites to nucleophilic attack by water molecules (Michael addition). Gerken et al. [113] partially suppressed Michael addition through methyl/hydroxy substitution and a per‐substitution strategy. However, under acidic conditions, degradation via a “protodesulfonation” pathway remained an issue. The Stahl group [114] employed a sulfonated thioether group to fully occupy and shield the reactive sites. Although this strategy came at the cost of a reduced redox potential, it significantly enhanced the molecule's cycling stability in acidic media. AQ derivatives, with their extended conjugated planar structure, exhibit lower membrane permeability and higher structural stability, making them the most extensively studied quinone‐based multi‐electron materials. Although the classic AQDS (9,10‐anthraquinone‐2,7‐disulfonic acid) demonstrates excellent performance, its reduced state is prone to disproportionation in alkaline media, generating electrochemically inactive anthrone and resulting in capacity fade. Specifically, the reduced dihydroxyanthraquinone molecule undergoes disproportionation, yielding one re‐oxidized AQDS molecule and one over‐reduced, inactive anthrone byproduct.

To address this issue, the Aziz group systematically introduced stabilizing substituents, such as hydroxy, alkoxy, and phosphonate groups (e.g., in 2,6‐DBEAQ, 2,6‐DPPEAQ, and DPivOHAQ), based on the AQDS framework. These modifications completely occupied potential nucleophilic attack sites, simultaneously improving solubility and significantly reducing the rates of disproportionation and dealkoxylation. This approach yielded ultra‐stable anolyte materials with an annual capacity decay rate of less than 1% under alkaline conditions.

Furthermore, introducing large cations such as tetramethylammonium (TMA^+^) into the electrolyte modulates the solvation structure of the reduced AQ^2^^−^ species. This increases the reaction steric hindrance, thereby suppressing the disproportionation side reaction at the molecular level and significantly extending battery cycle life.

**FIGURE 6:**
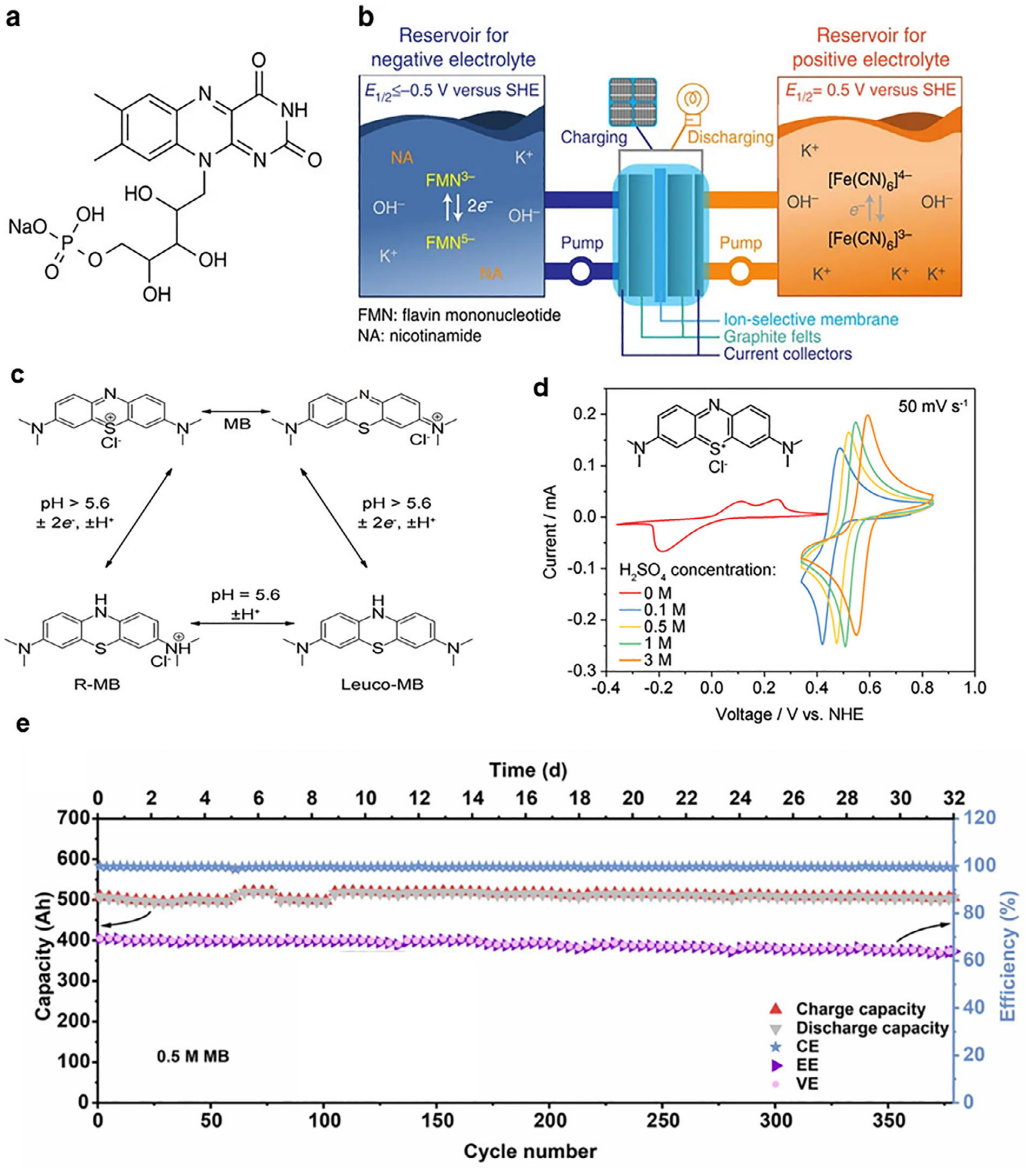
(a) The molecular structure of riboflavin‐5′‐monophosphate sodium salt (FMN‐Na, sodium salt of flavin mononucleotide). (b) Schematic representation of an RFB consisting of FMN‐Na and ferrocyanide‐based negative and positive electrolytes, respectively. Reproduced with permission [121]. Copyright 2022, American Association for the Advancement of Science. (c) pH‐dependent redox process of MB. (d) CV curves of MB at different H_2_SO_4_ concentrations; (e) The life cycle of the 0.5 M MB/V flow stack batteries. Reproduced with permission [118]. Copyright 2023, Royal Society of Chemistry.

Aza‐aromatic compounds, such as alloxazines, phenazines, and phenothiazines, contain imine‐like nitrogen atoms (─N═) within their molecular core structures. These nitrogen atoms serve as redox‐active centers, endowing such compounds with the potential for two‐electron transfer reactions. However, their practical application is still hindered by challenges, including limited electron transfer numbers and complex degradation mechanisms [115]. Among alloxazine derivatives, riboflavin‐5′‐phosphate sodium salt (FMN‐Na) is theoretically capable of undergoing two‐step redox reactions (Figure 6a, b). However, in practice, its application is constrained by a tendency toward dimerization and hydrolytic instability. Although the addition of nicotinamide as a hydrotropic agent can increase its solubility to 1.5 M, only partial two‐electron capacity is achieved. Moreover, dimer formation leads to a negative shift in redox potential, thereby reducing the theoretical voltage plateau [44]. Similarly, alloxazine‐7/8‐carboxylic acid (ACA) exhibits high solubility (2 M) in pH 14 KOH solution. Its deprotonated anionic structure effectively repels nucleophilic attack by OH^−^, slowing the degradation rate due to hydrolytic ring‐opening. Nonetheless, a capacity fade of 0.013% per cycle persists, indicating that full two‐electron reversibility has not yet been attained [116].

Investigation into the multi‐electron transfer performance of phenazine derivatives reveals more complex structure‐property relationships. Through an asymmetric substitution strategy, 7,8‐dihydroxyphenazine‐2‐sulfonic acid (DHPS) achieves both high solubility (1.8 M) and a low redox potential (–0.9 V vs. NHE). When paired with a ferrocyanide/ferricyanide positive electrode, it delivers a high volumetric capacity of 67 Ah L^−1^. However, studies indicate kinetic limitations in its two‐electron transfer process. Broadened NMR peaks of the reduced species suggest aggregation or coordination behavior in solution, which may affect long‐term cycling stability. More importantly, the degradation mechanisms of the phenazine framework are more complex than initially expected. Through systematic experimental and theoretical studies, Zhang et al. [117] discovered that amino‐acid‐functionalized phenazine derivatives undergo tautomerization‐based degradation. This process strongly depends on the position of the substituents: derivatives substituted at the 2,7‐ and 1,8‐positions, due to insufficient steric hindrance, are prone to dearomatization of the benzene ring, resulting in loss of redox activity. In contrast, placing functional groups at the 1,6‐positions effectively suppresses this degradation pathway via stereoelectronic effects, providing key guidance for designing truly stable two‐electron phenazine molecules.

Among phenothiazine derivatives, methylene blue (MB) represents a successful example of stable two‐electron redox behavior. Under acidic conditions (3 M H_2_SO_4_), it undergoes a concerted two‐electron redox process at a potential of 0.57 V versus NHE (Figure 6c, d), with a high kinetic rate constant of 0.32 cm s^−1^ [118]. This exceptional stability originates from the strong electron‐donating and conjugative effects of its two dimethylamino groups, which effectively delocalize the positive charge density on the sulfonium ion oxidation product, thereby suppressing nucleophilic attack by water molecules and hydrolytic side reactions. The recent successful demonstration of a kW‐scale flow battery stack based on MB and a vanadium negative electrode, which stably cycled for over 500 cycles at 80 mA cm^−^^2^ (Figure 6e), along with the confirmation of its high stability under practical operating conditions via in situ NMR and EPR techniques, provides strong evidence for the commercial viability of aza‐aromatic compounds [119].

The functionalization of traditional one‐electron systems offers a novel pathway for achieving multi‐electron activity. Yuan and Minteer [120] innovative design of a fluorenone derivative (4C7SFL) challenged the conventional wisdom that mono‐carbonyl compounds are difficult to utilize for reversible two‐electron reactions. A key finding of this study is that the storage of the second electron does not occur via the conventional direct electrochemical reduction pathway, which suffers from a high energy barrier due to the high pKa of the benzylic proton, but rather through a reversible chemical disproportionation reaction (2A^−^⇌A+A^2^^−^) of the radical anion generated by one‐electron reduction. This “electrochemical–chemical” coupling mechanism cleverly bypasses the high‐energy barrier associated with direct two‐electron transfer, offering a universal strategy for transforming a range of carbonyl and imine compounds traditionally limited to one‐electron reactions into multi‐electron systems. This significantly expands the structural diversity of molecules capable of multi‐electron activity.

### 3.5 Polymeric Multi‐Electron Systems

The design of polymeric active materials aims to overcome the crossover and cost limitations of traditional small‐molecule active species in redox flow batteries (RFBs) through macromolecular engineering [121, 122, 123]. In such systems, the multiple, often independent redox centers along the polymer chain can facilitate cooperative multi‐electron transfer across the entire molecule, as exemplified by copolymers of poly(viologen) and poly(TEMPO). However, their development remains constrained by challenges in energy density, cycle life, and kinetic performance. Winsberg et al. [124] pioneered an aqueous polymer RFB (pRFB) based on poly(viologen) and poly(TEMPO) copolymers, replacing expensive ion‐selective membranes with low‐cost size‐exclusion membranes. This system achieved a voltage of 1.1 V and an energy density of 10.8 Wh L^−^^1^, demonstrating the feasibility of suppressing crossover via macromolecular size effects. Nonetheless, the energy density was fundamentally limited by the solubility of the polymers in water. To break this constraint, Yan et al. [125] introduced zwitterionic comonomers to increase the capacity concentration of poly(TEMPO) to above 20 Ah L^−^^1^ while effectively managing viscosity. However, this approach did not address the inherent electrochemical window limitation of aqueous electrolytes (1.23 V).

Switching to nonaqueous systems can widen the voltage window, as demonstrated by Montoto et al. [126], who developed a poly(phthalimide)/poly(TEMPO) battery with a voltage as high as 2.2 V. Yet, such systems often suffer from severe capacity decay (40% loss after 10 cycles). Symmetric cell designs, inspired by all‐VFBs, can reduce irreversible capacity decay from active material crossover. One study used a BODIPY‐based polymer with bipolar redox activity as both positive and negative electrode material [127]. This polymer shows two reversible redox processes at −1.5 and 0.7 V (vs. Ag/Ag^+^), giving a theoretical voltage near 2.0 V. In practice, cells showed low Coulombic efficiency (<90%) and a sloped discharge curve, limiting energy efficiency. These results highlight the difficulty of maintaining polymer stability and reversibility in nonaqueous electrolytes while achieving higher operating voltages.

To completely circumvent solubility constraints and reduce solution viscosity, aqueous particulate polymer slurry batteries (APPSBs) based on insoluble polymer particles have emerged as a promising direction. Ding et al. [128] reported an all‐polymer particulate slurry battery using micron‐sized poly(hydroquinone) (PHQ) and polyimide (PI) particles, forming a stable low‐viscosity slurry with a high active material concentration of 1.0 mol L^−^^1^ in acidic electrolyte. Charge transport in this system depends on a unique “site‐hopping” mechanism: initial electron transfer occurs via particle‐electrode collisions, followed by charge propagation through densely distributed redox centers (e.g., quinone groups) along the polymer chains [39]. Although a capacity of 8.95 Ah L^−^^1^ was achieved, the capacity retention dropped to 70% after 300 cycles, primarily due to sluggish internal charge transfer kinetics (diffusion coefficient D ≈ 10^−^^7^ cm^2^ s^−^^1^) and particle aggregation. Experiments confirmed that reducing particle size from 5.6 to 0.9 µm via ball milling significantly enhanced kinetics and capacity utilization.

Increasing the number of electrons transferred per molecule (*n*‐value) to boost flow battery energy density faces several challenges. Multi‐step redox processes often show energy efficiency loss from separated potentials, unstable high‐valent intermediates, and slow kinetics. The nature of these challenges differs between stepwise and cooperative transfer pathways: stepwise processes require stabilization of reactive intermediates, while cooperative pathways aim to minimize potential separation for a flat discharge plateau. Limited solubility of multi‐electron materials further restricts practical capacity, caused by strong intermolecular π–π stacking, hydrogen bonding, and high lattice energy. Higher electrolyte concentration increases viscosity, raising mass transfer resistance and pumping losses that reduce power density and cycle life. Developing high‐concentration electrolytes addresses these limits by targeting the concentration term in energy density. Molecular designs for multi‐electron transfer, including the addition of hydrophilic groups or adjustment of steric hindrance, often improve solubility. This synergy between high n‐value and high concentration offers a combined route to next‐generation high‐energy‐density flow batteries.

## 4 High‐Concentration Electrolyte Systems

Developing high‐concentration electrolytes demands balanced solubility with mass transport. This requires overcoming intermolecular interactions like π–π stacking and electrostatic forces between active species. Low viscosity must also be preserved for efficient pumping. Current strategies primarily focus on molecular structural modification, solvation engineering.

### 4.1 Molecular Engineering

**FIGURE 7:**
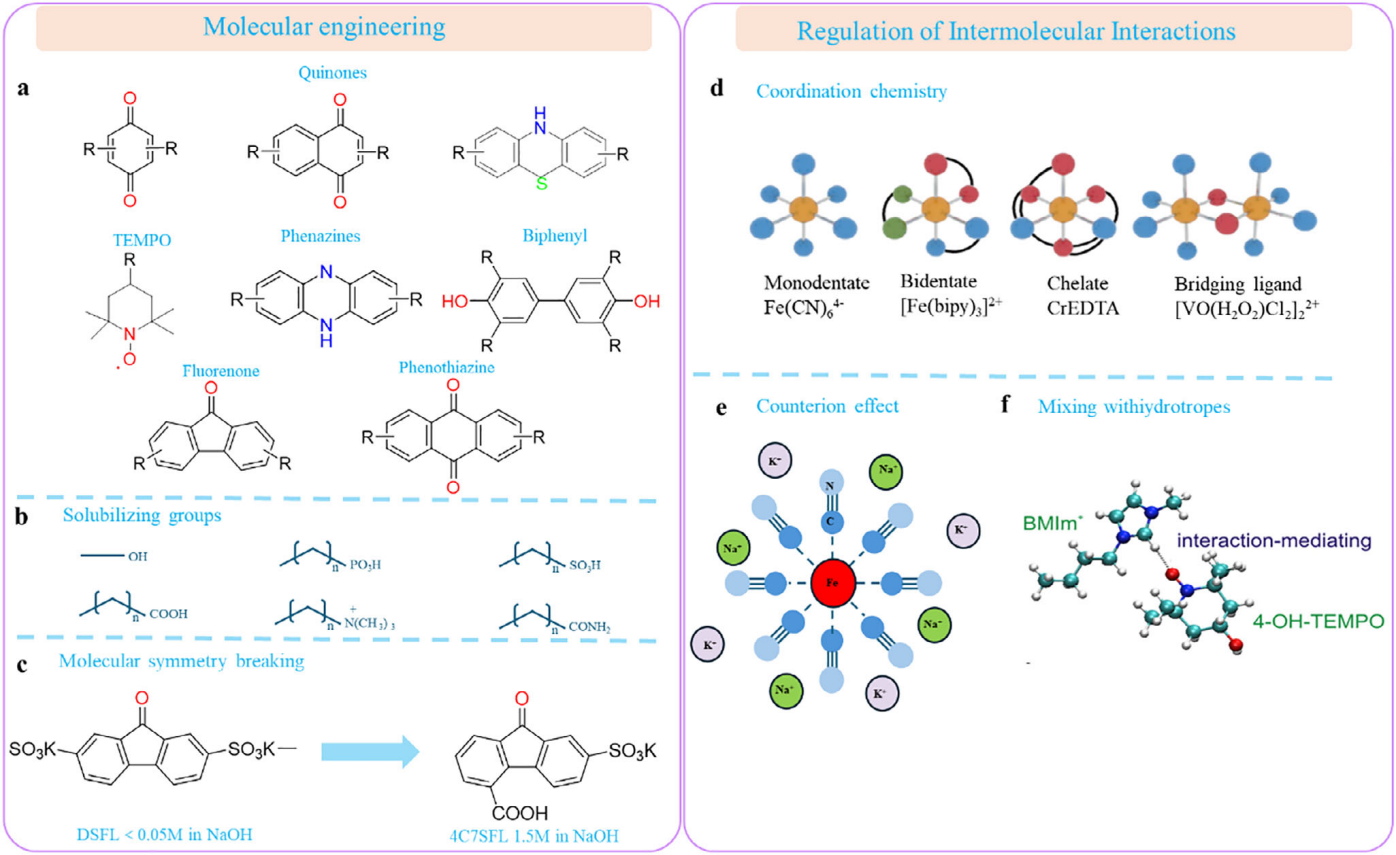
(a) Common molecularly engineered modification sites for aqueous organic reactive molecules, −R for functional group. Strategies to increase the aqueous solubility of redox‐active molecules. (b) Functional groups manipulation. (c) molecular symmetry breaking. (d) Coordination chemistry, classification of coordination compounds based on ligand binding modes. Reproduced with permission [140]. Copyright 2024, Springer Nature. (e) cation conditioning. (f) mixing with hydrotropes. Reproduced with permission [142]. Copyright 2020, Elsevier.

Among various molecular modification strategies, the introduction of strongly hydrophilic functional groups is widely regarded as the most direct and effective approach (Figure 7a) [129]. Functional moieties such as sulfonate, phosphate, quaternary ammonium, and carboxylate groups significantly enhance molecular compatibility with the aqueous phase through ionization and hydrogen‐bonding interactions (Figure 7b). Another strategy is breaking the molecule symmetry (Figure 7c). Rational molecular engineering of hydrophilic groups constitutes a core strategy for improving the solubility of organic electroactive species [130]. The underlying mechanisms involve enhanced polarity, ionization‐induced solvation, regulation of molecular conformation, and electrostatic stabilization. Precise integration of hydrophilic groups with tailored properties into specific molecular scaffolds effectively improves dissolution behavior and electrochemical performance.

Polyethylene glycol (PEG) chains substantially enhance solvation capability by forming dynamic hydrogen‐bond networks with polar solvents via ether oxygen atoms [72]. This approach has demonstrated remarkable success in viologen derivatives: PEG12‐V exhibits a solubility exceeding 2.5 M in acetonitrile, compared to a merely 0.12 M for unmodified tetramethyl viologen under identical conditions. Its matching molecule, a PEGylated phenothiazine derivative, also shows outstanding solubility, reaching 1.8 M, far surpassing the 0.05 M solubility of the unmodified phenothiazine. Nonaqueous flow batteries constructed from these materials demonstrate exceptional cycling stability, maintaining over 99.9% capacity retention after 300 cycles, with Coulombic efficiency consistently exceeding 99.8%. Quaternary ammonium groups provide permanent positive charges, generating strong hydration and electrostatic stabilization effects in aqueous environments. Incorporating a quaternary ammonium group into a ferrocene derivative (e.g., FcNCl) increases its aqueous solubility to 4.0 M, contrasting sharply with the 0.0012 M solubility of unmodified ferrocene. The bipolar molecule BTMAP‐Vi, modified with quaternary ammonium, achieves an aqueous solubility of 1.6 M. The symmetric cell system based on this molecule exhibits excellent cycling stability in neutral aqueous electrolyte, retaining 98.5% capacity after 1000 deep cycles [131]. Sulfonate groups can be fully ionized to negatively charged sulfonate ions under acidic conditions, forming highly hydrophilic molecular structures. Anthraquinone‐2,6‐disulfonic acid (AQDS) demonstrates a solubility of 1.0 M in 1.0 M sulfuric acid, vastly superior to the 0.0008 M solubility of unmodified AQ under the same conditions [132]. AQDS‐based flow battery systems maintain over 80% capacity utilization at current densities of 100 mA cm^−^^2^ and show no significant performance degradation over 500 cycles [133]. Carboxyl groups act as pH‐responsive moieties, ionizing into carboxylate anions under alkaline conditions and producing a pronounced hydrophilic effect [123]. Carboxyl‐functionalized 1,8‐dihydroxyanthraquinone reaches a solubility of 0.8 M in alkaline solution (pH > 10), significantly higher than the 0.015 M solubility of the unmodified analogue [134]. An alkaline flow battery employing carboxyl‐functionalized phenazine demonstrates 99.5% capacity retention over 200 cycles, indicating promising application potential [135].

Beyond conventional single‐charged modifications, zwitterionic molecular design integrating both cationic and anionic groups presents a distinct solubility enhancement strategy. Intramolecular charge separation confers a substantial dipole moment, driving strong ion‐dipole interactions with water and a favorable Gibbs free energy of solvation. Critically, zwitterions often exhibit an antipolyelectrolyte effect where solubility increases with salt concentration, attributable to ionic shielding of intramolecular electrostatic attractions that otherwise promote aggregation. This salt‐tolerant solubility directly enables innovative battery materials. The soft–hard zwitterionic trapper (SH‐ZIT) exemplifies this, combining a polyhalide‐complexing cation with a solubilizing sulfonate anion to achieve homogeneous cycling at 2 M concentration and > 90% state of charge with exceptional stability [96]. This approach extends to organic and inorganic flow battery systems. For instance, imidazolium‐/pyrrolinium‐substituted persistent zwitterionic ferrocenyl derivatives exhibit enhanced thermal stability and persistent solubility, crucial for long‐term cycling of organic electrolytes [136]. In a biomimetic design exemplifying the zwitterionic approach, an artificial redox‐active α‐amino acid molecule, synthesized by grafting cysteine side chains onto a 1,5‐dihydroxyanthraquinone core, demonstrates a substantial increase in aqueous solubility (0.63 M) compared to the parent molecule (0.2 M) and achieves an exceptional cycling stability with a capacity decay rate as low as 0.00025% per cycle. This performance is attributed to its unique zwitterionic structure and abundant hydrogen‐bonding interactions, which effectively suppress molecular dimerization and enhance solvation [137]. As hydrotropes or viscosity modifiers, zwitterionic molecules disrupt π–π stacking and radical dimerization, kinetically suppressing aggregation. Designing these functionalities requires balancing spacer length and charge group selection to optimize hydration without compromising redox activity. Despite added synthetic complexity, zwitterionic engineering provides a holistic approach to achieving high‐concentration, aggregation‐resistant electrolytes for next‐generation high‐energy‐density flow batteries.

Other hydrophilic groups beyond conventional types provide distinct advantages in specific applications. Phosphate groups maintain hydrophilicity over wide pH ranges through stepwise ionization. Their strong coordination capability benefits battery systems utilizing metal ion interactions. Hydroxy groups offer moderate hydrophilicity but significantly increase molecular polarity through multi‐hydroxyl synergy. These groups are also attractive for their synthetic accessibility and low cost [138]. Amide groups, possessing both hydrogen‐bond donor and acceptor capabilities, demonstrate good compatibility with various electrolyte solvents [139]. Furthermore, groups such as quaternary phosphonium salts and sulfonamides have also been utilized in specialized electrolyte environments owing to their distinctive electronic effects and solvation behaviors [140]. These less conventional hydrophilic groups provide additional options for molecular design to meet specific requirements, laying an important materials foundation for the development of next‐generation high‐performance flow battery systems [141].

### 4.2 Regulation of Intermolecular Interactions

Enhancing the solubility of active materials through the strategic utilization of mutual influences among different substances represents a powerful solubility‐enhancement strategy. The core of this approach lies in the precise design of intermolecular interactions that alter the solvation structure and physicochemical state of active species, thereby overcoming their intrinsic solubility limits [143, 144]. In contrast to complex molecular synthetic modifications, coordination chemistry(Figure 7d) offers a more efficient pathway by reconstructing the microenvironments of existing high‐capacity yet poorly soluble molecules.

In the design of aqueous flow battery systems, the selection of ligands capable of forming highly stable chelates (e.g., EDTA, DTPA, etc.) represents a critical strategy for achieving ultrahigh solubility [145]. This approach offers two principal advantages: first, the strong chelation effect provided by multidentate ligands significantly reduces the energy barrier for complex dissolution from the crystal lattice; second, the inherent hydrophilic groups on the ligands markedly enhance the overall hydration of the coordination complex. For instance, DTPA, leveraging its structural capacity for up to eight coordination sites, effectively suppresses the coordination of water and OH^−^, thereby inhibiting the formation of Fe(OH)_3_ precipitates [146]. As a result, K_2_FeDTPA achieves a solubility as high as 1.35 M at pH 9, corresponding to a remarkable capacity of 36.2 Ah L^−1^. Increasing ligand structural asymmetry and molecular conformational flexibility can inhibit orderly packing during crystallization and reduce precipitation tendency, thereby enhancing solubility by raising the system's mixing entropy. For example, replacing traditional K^+^/Na^+^ counterions in ferrocyanides with NH_4_^+^ or Li^+^ not only reduces lattice energy but also strengthens ion hydration (Figure 7e). Luo et al. [147] and Carvalho et al. [148] achieved concentrations of 1.6 M (NH_4_)_4_[Fe(CN)₆] and 2.3 M Li_4_Fe(CN)₆ in neutral solutions, respectively, resulting in volumetric capacities as high as 54 Ah L^−1^. A key advantage of this strategy is its ability to break through solubility limits without modifying the structure of the active molecule itself. Furthermore, the mixed‐ligand strategy enables synergistic coordination interactions to concurrently optimize both solubility and stability. In iron‐based battery electrolytes, a dual‐ligand system comprising TEA and 2‐methylimidazole increased the solubility of iron ions from approximately 0.8 M under single‐ligand conditions to about 1.4 M [149]. Even at such high concentrations, the system still exhibited excellent electrochemical stability, achieving a cycle life of over 1400 cycles. Similarly, in vanadium‐based flow batteries, the solubility limit of V(V) ions in conventional 3 M H_2_SO_4_ electrolyte is generally around 2.2 M, accompanied by poor stability at elevated temperatures [150]. By introducing hydrochloric or phosphoric acid to form mixed‐acid electrolytes, Cl^−^ or PO_4_^3−^ disrupts the hydrated structure of vanadium ions through competitive coordination, thereby enabling a stable increase in total vanadium concentration to over 2.7 M [151].

Polymeric polyelectrolyte additives represent an emerging approach in electrolyte design (Figure 7f). PEDOT:PSS colloids function as polyionic additives. Their surfaces carry abundant sulfonate groups that preferentially coordinate with Na^+^. This coordination disrupts the rigid supramolecular aggregates formed between organic active molecules like DHPS and Na^+^ ions [150]. Adding 1 wt% PEDOT: PSS to Na^+^‐containing quinone‐based electrolytes increases the apparent solubility of the active material from 0.8 to 1.2 M, a 50% enhancement, while reducing solution viscosity by approximately 15% and increasing ionic conductivity by about 4%. Such methodologies demonstrate effective pathways for achieving high‐concentration dissolution and transport optimization by precisely regulating solution microenvironments and ionic states.

## 5 Membranes

The preceding sections have discussed how voltage window expansion, multi‐electron transfer, and high‐concentration electrolytes can theoretically enhance energy density. In practice, however, achieving this theoretical potential places greater demands on other cell components. As pursued in Sections 2 through 4, high voltages and concentrated active species significantly increase the driving force for crossover and the chemical stress on the membrane, making the membrane a critical bottleneck. This section, therefore, examines recent advances in membrane materials as a technology designed to provide the selectivity, conductivity, and stability required for the systems described above.

High‐concentration electrolytes and wide voltage windows improve energy density, but they also intensify active species crossover and accelerate membrane degradation at extreme potentials [152, 153]. These issues present critical bottlenecks for practical performance. Therefore, optimizing membrane selectivity and stability serves as a critical enabler for implementing high‐energy‐density flow batteries [154, 155, 156]. Researchers often use highly soluble active species to create high‐concentration electrolytes, aiming for high theoretical energy density [121]. The steep concentration gradient, however, greatly increases the driving force for active species to cross the membrane, posing a major challenge to membrane selectivity. The performance of ion‐conducting membranes (ICMs) directly controls battery energy efficiency and long‐term capacity retention [157]. This relationship makes the membrane a critical bottleneck that limits the practical energy density of these systems [158, 159].

Conventional ion‐exchange membranes, including PFSA and sulfonated poly(ether ether ketone) (SPEEK), employ fixed charged groups (e.g., ─SO_3_^−^ or ─NR_3_^+^) to impart ion selectivity via Donnan exclusion [160]. These membranes often possess high ionic conductivity, which supports high voltage efficiency. However, they are prone to excessive swelling in high‐concentration electrolytes. The swollen state enlarges the internal ion channels, weakening the barrier against active species and causing severe crossover [161, 162]. This crossover directly lowers Coulombic efficiency and triggers irreversible capacity fade, ultimately restricting the battery's long‐term energy density. Suppressing membrane swelling and resolving the inherent conductivity‐selectivity trade–off are therefore central goals in membrane material development [131].

New membrane designs address these limitations of conventional membranes. Recent efforts have focused on modified ion‐exchange membranes and materials with new transport mechanisms. Amphoteric ion‐exchange membranes (AIEMs) contain both cationic and anionic functional groups [163]. This structure improves ion selectivity without sacrificing proton conductivity. For instance, AIEMs fabricated by blending quaternized poly(ether imide) (QAPEI) with SPEEK demonstrated a high Coulombic efficiency of 96.1% and energy efficiency of 88.5%, outperforming commercial Nafion membranes [164]. Ion‐solvating membranes (ISMs), like polybenzimidazole (PBI), use solvation‐coordination mechanisms for proton transport. These membranes show exceptional chemical stability and low crossover in acidic environments. An mPBI membrane treated with H3PO4‐assisted pre‐swelling exhibited low area‐specific resistance. In battery tests, this membrane enabled energy efficiencies of 78%–95% at current densities from 20 to 100 mA cm^−^^2^ [165].

Beyond functional modifications in AIEMs and ISMs, recent membrane designs target molecular‐level ion channel engineering to overcome the conductivity‐selectivity trade–off. This approach moves past traditional homogeneous polymers. One strategy constructs microphase‐separated channels via molecular self‐assembly, demonstrated by cost‐effective modified SPEEK membranes. In alkaline AORFBs using K_4_Fe(CN)_6_/DHAQ redox couples, SPEEK shows a very low K_4_Fe(CN)_6_ permeability of 5.3×10^−12^ cm^2^ s^−1^. This value is nearly two orders of magnitude lower than that of Nafion 212 (4.5×10^−10^ cm^2^ s^−1^). This high blocking capability, together with low area‐specific resistance, allows stable cycling at 250 mA cm^−^^2^ with excellent capacity retention. These properties make SPEEK suitable for high‐concentration electrolytes. Poly(2,6‐dimethyl‐1,4‐phenylene oxide) forms another key MSM platform. Using DABCO as a bifunctional cross‐linker produces QPPO membranes that deliver 99.3% Coulombic efficiency in TEMPO/Viologen systems [166, 167].

Another sophisticated strategy employs rigid polymer architectures to create intrinsic microporous channels with precise molecular sieving capabilities [168, 169, 170, 171, 172]. Polymers of Intrinsic Microporosity (PIMs) functionalized with amidoxime groups demonstrate this approach effectively [173]. These membranes maintain high ionic conductivity (21.5 mS cm^−^^1^ in 5.0 M KOH) while exhibiting extraordinary selectivity, with Dsol/Deff ratios of 3.4×10^4^ for TEMPO‐sulfate, enabling stable operation in Zn‐TEMPO‐sulfate cells with minimal capacity decay (0.06% per cycle over 50 cycles) [174]. Carboxylate‐functionalized PIMs (cPIMs) further refine this concept through strategic incorporation of aryl segments to optimize pore hydrophobicity. The cPM‐Ph variant, with precisely tuned pore gates of 5.0±0.3 Å, achieves unprecedented ferrocyanide permeability of ∼4×10^−13^ cm^2^ s^−1^ while maintaining high ionic conductivity (14.6 mS cm^−^^1^), supporting exceptional cycling stability in 2,6‐D2PEAQ/ferrocyanide AORFBs with decay rates as low as 0.014% per day [175]. Tröger's Base membranes constitute another important intrinsic microporous membrane (MPM) family, where hyperbranched quaternized TB framework membranes (Q‐TBF‐TPB) combine high IEC (2.09 mmol g^−^^1^) with robust mechanical stability, delivering low area resistance (0.53 Ω cm^2^) and outstanding power density (182 mW cm^−^^2^) in TEMPTMA/BTMAP‐Vi systems with stable operation exceeding 1000 cycles [176].

Building on the advantages of microphase‐separated and intrinsic microporous channels, the most cutting‐edge innovations involve designing membranes with dual ion channels that synergistically combine the advantages of both microphase‐separated and microporous structures. Cross‐linked poly(aryl piperidinium) membranes (MTCP‐50) exemplify this approach, simultaneously forming microphase‐separated channels (d‐spacing ∼2.0 nm) and intrinsic micropores (∼5.2 Å diameter). This dual‐channel architecture enables high Cl^−^ conductivity (29 mS cm^−^^1^) while achieving exceptional performance in TEMPTMA/MV AORFBs with peak power density reaching 313.7 mW cm^−^^2^ [177]. Hypercrosslinked PPO membranes (HC‐QPPO) created through Friedel–Crafts alkylation transform conventional QPPO into microporous networks, reducing BTMAP‐Vi permeability to 3.40×10^−^^1^ ^1^ cm^2^ s^−1^, an order of magnitude improvement over pristine QPPO, while supporting outstanding cycling stability (> 1800 cycles) in 0.5 M TEMPTMA/BTMAP‐Vi cells with minimal capacity fade (0.0017% per cycle) [178]. Branched TB membranes (b‐TB) push these boundaries further by achieving high IEC (1.3 mmol g^−^^1^) combined with branched topology, creating both narrow microphase‐separated channels (2.0 nm d‐spacing) and micropores (2.3 Å radius) that collectively enable ultra‐low permeability (10^−^^1^ ^2^ cm^2^ s^−^^1^ level) and record‐breaking energy efficiencies in FcNCl/BTMAP‐Vi AORFBs.

**FIGURE 8:**
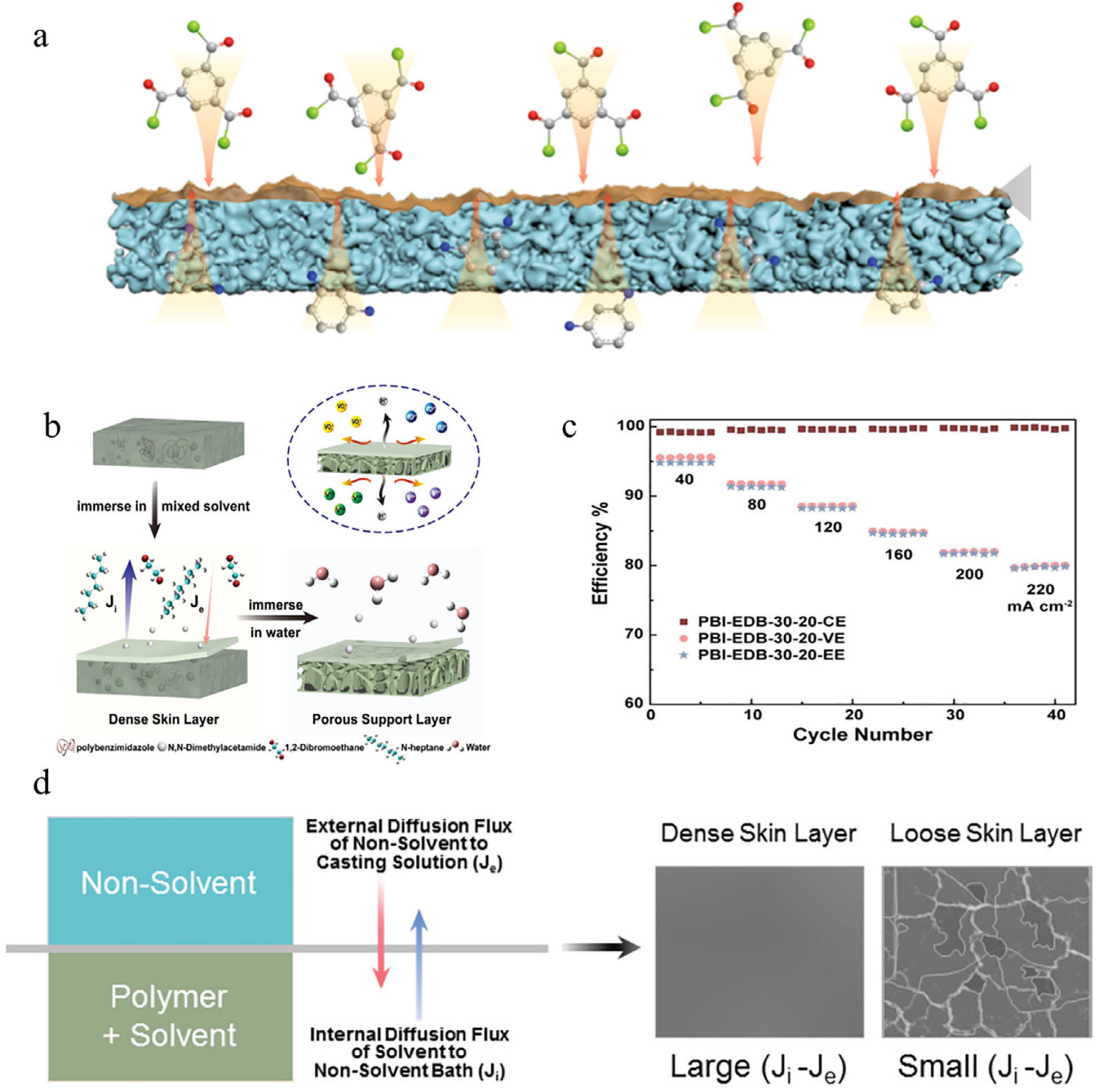
(a) Schematic diagram of PA‐based TFCM. Reproduced with permission [179]. Copyright 2020, Springer Nature. (b) Schematic diagram of the two‐step NIPS method. (c) VFB performances of the as‐prepared membrane at different current densities. (d) The cross‐section morphology of the as‐prepared membrane. Reproduced with permission [182]. Copyright 2020, WILEY.

To translate these molecular‐level design philosophies into practical, scalable membranes, researchers have developed various types of composite membranes through structural design, aiming to overcome this trade–off limitation and pave the way for high‐energy‐density flow batteries. Unlike homogeneous membranes, composite membranes typically consist of a functional “selective layer” and a mechanically supportive “support layer.” This structure allows for independent optimization of membrane selectivity and conductivity. By making the highly selective functional layer extremely thin, the resistance to ion transport can be minimized, while a highly porous and stable support layer ensures rapid carrier transport and mechanical integrity of the membrane. Polymer‐based thin‐film composite membranes (TFCMs) achieve excellent performance by constructing a dense or microporous polymer selective layer on a porous substrate. The core strategy lies in precisely controlling the internal structure of the selective layer to build efficient transport channels. Dai et al. [179] prepared a polyamide (PA) selective layer with a thickness of only 180 nm on a porous substrate via interfacial polymerization, as shown in Figure 8a. The PA network formed channels with sizes ranging from 3.6 to 6.2 Å, which are significantly smaller than the diameter of hydrated vanadium ions (> 8 Å) but allow protons (< 4.8 Å) to pass through. This enabled effective blocking of vanadium ions and highly efficient proton conduction. The VFB equipped with this membrane achieved an energy efficiency exceeding 80% even at a high current density of 260 mA cm^−^^2^, demonstrating outstanding performance. Kwabi et al. [180] utilized the Langmuir–Blodgett (LB) technique to prepare a highly ordered PFSA selective layer with a thickness of only 42 nm. The ultrathin thickness endowed the membrane with extremely low area‐specific resistance, while its highly ordered dense structure provided ion selectivity 500 times higher than that of conventional Nafion membranes, enabling stable operation for over 800 cycles. Introducing inorganic nanoparticles into the selective layer can further enhance membrane selectivity and stability by leveraging their regular crystal structures, rigid channels, and unique physicochemical properties. In VFBs, zeolite molecular sieves with precise pore sizes serve as ideal materials for ion sieving. Yuan et al. [181] constructed a selective layer using ZSM‐35 zeolite nanosheets with a pore size of 0.5 nm on the surface of a porous membrane. This pore size effectively intercepted hydrated vanadium ions while allowing protons to pass freely, achieving an H^+^/VO^2^^+^ selectivity as high as 44,300, three orders of magnitude higher than that of commercial Nafion 115 membranes. To further enhance the versatility of composite membranes and promote sustainability, recent studies have integrated biomaterials into polymer matrices.

Despite the excellent performance of TFCMs, issues such as poor interfacial compatibility and high interfacial resistance often exist between the selective layer and the support layer. To solve this challenge, Shi et al. [182] developed a “two‐step nonsolvent induced phase separation (NIPS)” method. By first immersing the casting solution in a nonsolvent immiscible with the solvent to form a dense skin layer, and then immersing it in a nonsolvent miscible with the solvent to form a highly porous support layer, they achieved independent control over the membrane structure (Figure 8b). The prepared membrane featured both a dense selective layer approximately 2.5 µm thick and a highly porous support layer (Figure 8c). The VFB assembled with this membrane achieved an energy efficiency exceeding 80% at a current density of 220 mA cm^−^^2^ (Figure 8d).

In summary, optimizing ion transport in RFB membranes is critical for improving energy and power densities. The design of membrane materials has evolved from traditional homogeneous polymers to structurally well‐defined and functionally integrated advanced composite frameworks and microporous materials. The frontier of membrane research now lies in the precise engineering of ion channels—whether through microphase separation, intrinsic microporosity, or a combination of both—to simultaneously achieve high conductivity and exceptional selectivity. These innovations are not merely ancillary improvements but are fundamental to unlocking the full potential of high‐energy‐density strategies, as they directly address the core challenge of active species crossover under demanding operating conditions. These innovations not only offer effective pathways to overcome the performance limitations of conventional flow batteries but also lay the foundation for developing next‐generation high‐energy‐density systems (e.g., zinc‐based and organic systems), enabling their commercial‐scale deployment in energy storage applications.

## 6 Challenges and Perspectives

### 6.1 Challenges

Enhancing energy density represents the central challenge limiting the widespread and cost‐effective commercialization of RFB technology. This review systematically examines three core strategies derived from the energy density equation: expanding the cell voltage, enabling multi‐electron transfer processes, and increasing the concentration of active species. Numerous molecular engineering strategies have demonstrated effectiveness in improving energy density. Principal approaches focus on designing high‐potential catholytes and low‐potential anolytes, developing multivalent inorganic and multi‐electron organic systems, and realizing high‐concentration electrolytes via solvation engineering [183]. For example, incorporating electron‐withdrawing groups can effectively modulate molecular orbital energy levels, leading to a positive shift in redox potential and correspondingly higher cell voltage. Simultaneously, introducing strongly hydrophilic moieties disrupts intermolecular forces, significantly improving solubility in aqueous media. Together, these strategies address both thermodynamic and kinetic aspects of the energy density equation, offering a multidimensional pathway for enhancing overall RFB performance.

Despite notable progress, translating laboratory innovations into commercial applications requires overcoming several key challenges:

1. *Balancing Performance and Stability*: The pursuit of high energy density frequently compromises stability, particularly when operating at extreme potential or implementing multi‐electron reactions [184]. Molecules at highly polarized potential often exhibit increased chemical reactivity, making them vulnerable to degradation during cycling. Likewise, multi‐electron transfer processes can generate thermodynamically unstable high‐valence intermediates, which are prone to disproportionation or irreversible side reactions, resulting in rapid capacity decay.
2. *System Integration Challenges*: Implementing high‐energy‐density electrolytes in practical systems introduces engineering difficulties. High‐concentration electrolytes generally lead to elevated viscosity, which increases pumping losses and severely restricts mass transport, thereby limiting power density and energy efficiency [185]. Furthermore, the combination of high voltage, high concentration, and novel active materials imposes exceptional requirements on critical components. Ion‐exchange membranes should achieve an optimal balance between high selectivity and ionic conductivity, a requirement that challenges existing materials. Additionally, the inherently slow reaction kinetics of many high‐energy‐density reactions at electrode surfaces present another major constraint on practical energy output.
3. *Cost and Scalability*: Many high‐performance organic active materials depend on complex, multi‐step syntheses, leading to high purification costs and limited production yields. This inherent conflict between performance and expense often renders laboratory‐demonstrated solutions commercially impractical. For large‐scale deployment of high‐energy‐density RFBs, it is essential to develop active materials that combine high performance with low cost, and that can be produced through simple, environmentally benign synthetic pathways.

### 6.2 Perspectives

Building upon the strategies to enhance energy density through voltage window expansion, multi‐electron transfer, and high‐concentration electrolytes, the transition from promising materials to reliable, high‐performance devices requires addressing critical challenges at both the component and system levels. The successful commercialization of high‐energy‐density RFBs depends not only on advances in active materials but equally on the systematic optimization of all core cell constituents: the electrolyte systems that store energy, the electrodes that mediate redox reactions, and the membranes that govern ionic selectivity. These elements form an interdependent system wherein the performance of each component directly dictates overall device functionality. Future research must therefore evolve from compartmentalized material development toward holistic system‐level optimization, carefully balancing the inherent trade‐offs between conductivity, selectivity, stability, and cost. This section outlines forward‐looking research perspectives for these three critical areas, beginning with the electrolyte as the central energy storage medium, followed by the electrodes and membranes as key functional components. Specifically, electrolyte formulations must reconcile high energy density with practical transport properties such as viscosity and ionic conductivity. Electrodes require designs that ensure efficient mass transport and interfacial stability while maintaining high electronic conductivity and catalytic activity [186]. Membranes need to achieve an optimal balance between low area‐specific resistance and high ion selectivity, coupled with long‐term durability under demanding electrochemical conditions.

#### 6.2.1 Electrolytes

As the central medium for energy storage, electrolytes directly determine the energy density ceiling and long‐term operational stability of RFBs. Future advancements must transcend the pursuit of high solubility to address the multifaceted challenges arising from ultra‐high concentrations, complex multi‐redox chemistries, and system integration. The following key research directions are critical for next‐generation electrolyte development:

1. *Decoupling Concentration from Transport Limitations*: The fundamental trade–off between high active‐material concentration and manageable viscosity remains a primary bottleneck. As exemplified by six‐electron transfer molecules like HATNTA, viscosity can exceed 30 mPa·s even at moderate concentrations (0.25 M), leading to severe pumping losses and mass transport limitations that compromise power density and capacity retention. Future efforts should focus on innovative thermodynamic and hydrodynamic strategies to break this linkage. Promising approaches include designing “localized high‐concentration electrolytes” where a dense solvation shell is maintained around active species while the bulk solution retains low viscosity, and employing advanced hydrotropes or dynamic rheological modifiers that disrupt unfavorable intermolecular interactions (e.g., π–π stacking, hydrogen‐bond networks) without sacrificing solubility. Achieving practical volumetric capacities of 40–60 Ah·L^−^^1^ necessitates such fundamental breakthroughs in electrolyte fluid engineering.
2. *Managing Dynamic Stability Across Multiple Redox States*: Multi‐electron organic active species undergo significant changes in solubility, aggregation propensity, and chemical reactivity as they cycle through different oxidation states. For instance, the fully reduced state of viologens exhibits markedly lower solubility than its oxidized form, creating a risk of precipitation and flow‐channel blockage under high‐concentration operation. Electrolyte engineering must, therefore, evolve from static formulation to dynamic stabilization. This involves tailoring supporting electrolytes (e.g., ionic strength, specific salt effects) and functional additives (e.g., solubilizing agents, radical scavengers) to modulate the solvation environment and suppress undesirable aggregation or disproportionation reactions in real‐time. Advanced operando spectroscopic techniques (e.g., NMR, EPR) are indispensable for elucidating these transient states and guiding rational electrolyte design.
3. *Engineering pH‐Asymmetric and Chemically Resilient Systems*: The aqueous electrochemical window and molecular stability are intrinsically pH‐dependent, creating a “trilemma” for matching high‐potential catholytes and low‐potential anolytes within a single pH environment. A paradigm‐shifting direction is the development of pH‐asymmetric RFB conFigureurations enabled by bipolar membranes (BPMs). This architecture allows the anolyte and catholyte to operate in their respective optimal pH regimes (e.g., alkaline for anolytes, acidic for catholytes), dramatically expanding the pool of stable organic molecules. Concurrently, molecular design must incorporate hydrolytically robust motifs for acidic conditions and oxidation‐resistant structures for alkaline environments. For neutral systems, which offer material compatibility advantages, dedicated research on high‐performance multi‐electron pairs and buffered supporting electrolytes is essential to mitigate pH drift during operation.
4. *AI‐Driven Formulation Optimization and Multi‐Objective Design*: The multivariate nature of electrolyte formulation—balancing concentration, viscosity, redox potential, chemical stability, and cost—presents a combinatorial challenge ideal for artificial intelligence (AI) and machine learning (ML). Beyond virtual screening for molecular properties, AI's greatest impact may lie in multi‐objective optimization of complete electrolyte recipes. ML models can integrate data from molecular descriptors, solvent properties, and additive effects to predict overall electrolyte performance and identify Pareto‐optimal formulations that balance competing requirements. Coupling this computational guidance with high‐throughput robotic experimentation will establish a closed‐loop “design‐make‐test” cycle, accelerating the discovery of electrolytes that are not only high in energy density but also tailored for system‐wide durability and efficiency.

#### 6.2.2 Electrodes

Future electrode development should focus on the rational design of porous architectures and surface properties to address kinetic and mass transport limitations:

1. *Precise Construction of Porous Structures*: Adopting hierarchical/gradient pore structures or three‐dimensional ordered macroporous electrodes (e.g., inverse opal structures) can homogenize electric field distribution and ion transport pathways, reduce concentration polarization, suppress dendrite growth, and retain high active material loading density. Such designs optimize electrolyte penetration and mass transfer efficiency, while increasing electrode specific surface area and porosity to ensure full exposure of active sites [187, 188]. Regulating pore size distribution and channel connectivity further enables efficient transport networks, laying the foundation for high‐power‐density operation.
2. *Surface Engineering and Catalytic Enhancement*: Beyond porous structure optimization, surface engineering is critical for boosting catalytic activity. Atomic‐level modification of carbon electrodes via heteroatom doping (e.g., N, O, S), defect engineering, and integration of low‐cost, high‐activity nonprecious metal composites can systematically enhance intrinsic electrocatalytic activity toward organic redox species (e.g., quinones, nitroxyl radicals), thereby reducing reaction overpotentials [189, 190]. Precise regulation of surface functional groups optimizes electrode wettability and interfacial compatibility, while modulating electronic structures to enhance electric double‐layer capacitance. These synergistic surface modifications significantly accelerate reaction kinetics, offering new pathways for efficient energy conversion.
3. *Cross‐Scale Collaborative Design*: To integrate structural and surface advantages, cross‐scale collaborative design is essential. Combining advanced imaging techniques (e.g., x‐ray tomography) with multi‐physics modeling can deeply reveal mass transport‐reaction coupling mechanisms under practical operating conditions, guiding rational electrode design spanning nanoscale active sites to macroscopic pore networks. Multi‐scale simulations accurately predict the impact of structural parameters on performance, providing a theoretical basis for optimizing electrode composition and conFigureuration. Establishing correlation models among material structure, interface characteristics, and battery performance enables targeted design and performance regulation, driving the development of next‐generation high‐performance electrodes.
4. *Integrating AI for Electrode Design*: Recent progress in ML and AI offers powerful tools to accelerate electrode material discovery and optimization for RFBs. For instance, topology optimization combined with multi‐physics models has been used to inversely design three‐dimensional porous electrodes, reducing overpotential by 29% and hydraulic power dissipation by 98% [191]. Similarly, genetic algorithms coupled with pore‐network models have optimized electrode microstructures, improving fitness by 75% while reducing pumping power by 73% [192]. By integrating AI‐driven materials databases with high‐throughput electrochemical simulations, it becomes possible to rapidly screen redox‐active molecules, catalysts, and electrode surface treatments to optimize key parameters such as redox potential, solubility, and stability. Furthermore, data‐driven approaches can uncover hidden correlations between molecular structures and electrochemical performance, guiding the rational design of novel organic and inorganic redox couples. A key remaining challenge is the seamless integration of these discrete scale‐specific models into a unified computational framework that can predict full‐cell performance. Looking forward, coupling AI models with autonomous experimental platforms and advanced manufacturing techniques holds the potential to significantly accelerate the development cycle of high‐performance electrode materials.

#### 6.2.3 Membranes

Complementary to electrode optimization, membrane advancement is equally critical for resolving the core trade–off between ion selectivity and conductivity, two key performance metrics constrained by the interdependent nature of cell components (electrode‐membrane‐electrolyte) highlighted in Section 6.2. Membrane innovation must align with electrode structure and electrolyte properties to achieve holistic system performance. Future research should focus on the following cutting‐edge directions:

1. *Precision Pore Structure Design*: Molecular‐level precision in pore architecture is the key to efficient ion sieving for membranes. Materials such as covalent organic frameworks (COFs), PIMs and engineered zeolites, featuring well‐defined pore geometries and tunable surface chemistry, provide an ideal platform for precise recognition. By accurately regulating pore size and surface functional groups, efficient discrimination of ionic species can be achieved via dual mechanisms of size exclusion and chemical affinity, thereby enhancing membrane ion selectivity.
2. *Advancement of Dynamic Smart Membranes*: Beyond static pore design, dynamic responsiveness represents the next frontier in membrane technology. Next‐generation membranes should transcend traditional static separation to stimuli‐responsive smart systems. Such materials can dynamically adjust pore size, surface charge, or hydrophilicity‐hydrophobicity in response to operational conditions (e.g., voltage, pH, ion concentration), mimicking the environmental responsiveness of biological ion channels. This adaptive mechanism enables real‐time optimization of ion transport pathways during battery operation, balancing selectivity and conductivity under varying working conditions.
3. *Multifunctional Integrated Design*: For metal‐based flow battery systems, developing multifunctional membranes is imperative, combining high ion selectivity, excellent mechanical integrity, and long‐term chemical stability. These membranes must effectively suppress dendrite penetration while maintaining mechanical integrity in highly corrosive electrolyte environments. Incorporating self‐healing capabilities can further enhance durability by enabling in situ repair of microdamage, delaying performance decay and laying a material foundation for the long cycle life of high‐energy‐density flow batteries.
4. *System‐Level Synergistic Optimization*: Finally, membrane development cannot be isolated from the overall battery system, synergy with electrolytes and electrodes is essential for practical application. By optimizing interfacial compatibility and electrochemical matching among components, an integrated battery system solution can be constructed, facilitating the transition of high‐energy‐density flow batteries from laboratory research to practical applications.
5. *AI‐Assisted Membrane Design and Optimization*: AI‐Assisted Membrane Design and Optimization: The design of ICMs requires navigating the fundamental trade–off between ion selectivity and conductivity. AI and ML are being explored to optimize this balance by guiding material synthesis and processing. For instance, ML models (linear regression and artificial neural networks) have been employed to screen solvent treatments for polybenzimidazole (PBI) porous membranes, achieving high prediction accuracy for voltage and energy efficiency with less than 1% mean absolute percentage error, and identifying alcohols as the optimal solvent class [193]. Beyond post‐processing optimization, AI can also guide the creation of novel membrane structures. In a recent study, a multi‐layer perceptron (MLP) model was used to inversely optimize the spin‐coating parameters for fabricating bioinspired, aligned graphene oxide membranes, leading to an exceptional ion selectivity of 21.60 × 10^4^ S min cm^−3^, which represents a tenfold improvement over pristine membranes, and stable cycling for over 5000 cycles [194]. These examples illustrate the potential of ML to transform membrane development from empirical approaches to more rational, data‐informed design. Despite these promising results, challenges such as accurately predicting long‐term chemical stability under dynamic flow battery conditions and scaling up computationally designed structures persist. Future efforts should focus on integrating physics‐informed AI models with multi‐scale simulations and high‐throughput experimental platforms to accelerate the discovery of durable membranes for next‐generation RFBs.

All membrane design strategies must be compatible with high‐concentration electrolytes (Section 4) and electrode surface properties. For instance, smart membranes should adapt to the viscosity and ion concentration of electrolytes, while multifunctional membranes must resist corrosion in aggressive electrolyte environments. This cross‐component compatibility is the core of system‐level optimization for high‐energy‐density flow batteries.

## 7 Conclusion

The development of high‐energy‐density flow batteries remains constrained by intrinsic trade–offs between energy density and operational stability, urgently requiring solutions to key challenges, including material degradation under extreme potential conditions, sluggish kinetics in multi‐electron reactions, and severe mass transfer limitations in high‐concentration electrolytes. While molecular orbital engineering has successfully realized significant improvements in operating voltage and multi‐electron reaction systems demonstrate potential for breaking theoretical capacity limits, these technological advances often lead to compromised cycling stability owing to intensified side reactions. Current research faces persistent challenges, including the inverse correlation between ionic conductivity and viscosity in high‐concentration electrolytes which severely limit power density, the instability of reactive intermediates during multi‐electron transfer processes that hinders cycle life improvement, and the chemical degradation of materials under high‐potential conditions that compromises long‐term operational stability. To develop practically viable high‐energy‐density systems, advanced in situ characterization techniques and multi‐scale simulations must be utilized to deeply elucidate reaction mechanisms and degradation pathways at the electrode‐electrolyte interface. Guided by structure‐property relationships, rational material design coupled with synergistic innovations in highly selective membranes and mass‐transfer‐optimized architectures will be crucial for advancing the practical implementation of flow batteries in large‐scale energy storage applications.

## Conflicts of Interest

The authors declare no conflicts of interest.
